# Proteogenomic integration reveals therapeutic targets in breast cancer xenografts

**DOI:** 10.1038/ncomms14864

**Published:** 2017-03-28

**Authors:** Kuan-lin Huang, Shunqiang Li, Philipp Mertins, Song Cao, Harsha P. Gunawardena, Kelly V. Ruggles, D. R. Mani, Karl R. Clauser, Maki Tanioka, Jerry Usary, Shyam M. Kavuri, Ling Xie, Christopher Yoon, Jana W Qiao, John Wrobel, Matthew A. Wyczalkowski, Petra Erdmann-Gilmore, Jacqueline E. Snider, Jeremy Hoog, Purba Singh, Beifang Niu, Zhanfang Guo, Sam Qiancheng Sun, Souzan Sanati, Emily Kawaler, Xuya Wang, Adam Scott, Kai Ye, Michael D. McLellan, Michael C. Wendl, Anna Malovannaya, Jason M. Held, Michael A. Gillette, David Fenyö, Christopher R. Kinsinger, Mehdi Mesri, Henry Rodriguez, Sherri R. Davies, Charles M. Perou, Cynthia Ma, R. Reid Townsend, Xian Chen, Steven A. Carr, Matthew J. Ellis, Li Ding

**Affiliations:** 1 Department of Medicine, Washington University in St. Louis, St. Louis, Missouri 63108, USA; 2 McDonnell Genome Institute, Washington University in St. Louis, St. Louis, Missouri 63108, USA; 3 The Broad Institute of MIT and Harvard, Cambridge, Massachusetts 02142, USA; 4 Department of Biochemistry & Biophysics, University of North Carolina, Chapel Hill, North Carolina 27599, USA; 5 Center for Health Informatics and Bioinformatics, New York University School of Medicine, New York, New York 10016, USA; 6 Lineberger Comprehensive Cancer Center, University of North Carolina, Chapel Hill, North Carolina 27599, USA; 7 Lester and Sue Smith Breast Center, Baylor College of Medicine, Houston, Texas 77030, USA; 8 Department of Pathology and Immunology, Washington University in St. Louis, St. Louis, Missouri 63108, USA; 9 Department of Genetics, Washington University in St. Louis, St. Louis, Missouri 63108, USA; 10 Department of Mathematics, Washington University in St. Louis, St. Louis, Missouri 63108, USA; 11 Verna and Marrs McLean Department of Biochemistry and Molecular Biology, Baylor College of Medicine, Houston, Texas 77030, USA; 12 Siteman Cancer Center, Washington University in St. Louis, St. Louis, Missouri 63108, USA; 13 Department of Anesthesiology, Washington University in St. Louis, St. Louis, Missouri 63108, USA; 14 National Cancer Institute, National Institutes of Health, Bethesda, Maryland 20892, USA

## Abstract

Recent advances in mass spectrometry (MS) have enabled extensive analysis of cancer proteomes. Here, we employed quantitative proteomics to profile protein expression across 24 breast cancer patient-derived xenograft (PDX) models. Integrated proteogenomic analysis shows positive correlation between expression measurements from transcriptomic and proteomic analyses; further, gene expression-based intrinsic subtypes are largely re-capitulated using non-stromal protein markers. Proteogenomic analysis also validates a number of predicted genomic targets in multiple receptor tyrosine kinases. However, several protein/phosphoprotein events such as overexpression of AKT proteins and ARAF, BRAF, HSP90AB1 phosphosites are not readily explainable by genomic analysis, suggesting that druggable translational and/or post-translational regulatory events may be uniquely diagnosed by MS. Drug treatment experiments targeting HER2 and components of the PI3K pathway supported proteogenomic response predictions in seven xenograft models. Our study demonstrates that MS-based proteomics can identify therapeutic targets and highlights the potential of PDX drug response evaluation to annotate MS-based pathway activities.

## Abstract (web summary)

Patient-derived xenografts recapitulate major genomic signatures and transcriptome profiles of their original tumours. Here, the authors, performing proteomic and phosphoproteomic analyses of 24 breast cancer PDX models, demonstrate that druggable candidates can be identified based on a comprehensive proteogenomic profiling.

Profiling of somatic alteration by next-generation DNA sequencing (NGS) has entered clinical practice with the promise of rapid diagnosis of druggable somatic genomic alterations for personalized cancer treatment^1^,^2^. For example, recent analysis of 4,068 samples from 16 cancer types suggested that repurposing approved drugs based on genomic alterations could provide individualized treatment options for around 40% of tumours^3^. However, clinical evidence for this proposition is limited and has been slow to develop. Further, the signalling and biological effects of somatic mutations are not routinely determined in human tumour samples even though this is a consideration for rational drug design, response prediction and target prioritization^4^,^5^. Finally, druggable genomic alterations are not detected in the majority of cases tested by NGS^3^. Comprehensive proteomic analyses provide a potentially valuable approach to validate genomic findings as likely biological drivers and to discover discover opportunities for targeted treatment.

Patient-derived xenografts (PDXs) in immunodeficient mice maintain the histological and molecular heterogeneity of the progenitor human tumour^6^ and cytotoxic drug responsiveness is often a transplantable phenotype^7^. Our previous studies have shown that breast tumour PDXs recapitulate major genomic signatures and transcriptome profiles of their original breast tumours^8^,^9^. Moreover, drug responses to endocrine therapy in breast cancer PDXs resembled that observed in the corresponding patient and endocrine therapy resistance patterns were associated with aberrations in the *ESR1* gene^6^. While comprehensive proteomic characterization of PDX is still lacking, recent studies using reverse phase protein array have identified similar protein profiles between PDX and primary tumours^10^,^11^. These studies collectively suggest that the PDX approach is a potentially valuable preclinical model for identification and testing of therapeutic targets.

Recent advances in mass-spectrometry provide an unprecedented opportunity for antibody-independent proteome profiling with approximately 80% of all proteins in major human tissues quantifiable by this technique^12^. Analyses of the global proteome in tandem with genomic profiles generated through TCGA have yielded key insights on the molecular etiology of colorectal cancer^13^, breast cancer^14^ and ovarian cancer^15^. Our recent proteogenomic characterization of 105 human breast cancers linked 5q-loss to elevated EGFR expression and identified overexpression of phosphorylated kinases including CDK12, PAK1 and ARAF as potential biological drivers^14^. However, current proteogenomic analyses of human tissues are limited by the fact that therapeutic hypotheses were not being addressed in these initial analyses. As patient treatments were heterogeneous, links between treatment and proteogenomic findings are difficult to establish in banked tumour specimens. The PDX system therefore provides an early opportunity to determine whether a proteogenomic approach can produce more robust therapeutic hypotheses and predictive biomarkers for individual patients than that achieved by genomic analysis alone.

In this study, we generated high-coverage proteome and phosphoproteome data for 24 PDX models representing different breast cancer subtypes using both isobaric mass-tag labelling and label-free proteomic methods for cross-validated identification of 12,794 proteins and 56,874 phosphorylation sites. After filtering for observation in at least 10 out of 24 samples 10,069 proteins and 36,609 phosphorylation sites with their relative abundances quantified across tumours, were used in subsequent analyses in this study. A substantial number of druggable protein/phosphoprotein events were uniquely identified. Further, similar proteomic/phosphoproteomic signatures were observed in patient breast cancer samples indicating potential clinical relevance. In selected PDX models exhibiting proteomic and/or phosphoproteomic up-regulation events of HER2 or the PI3K pathway, we validated the efficacy of targeted inhibitors in suppressing PDX growth. Taken together, these observations show that comprehensive proteogenomic profiling has potential to identify new targets for individualized treatment approaches in cancer.

## Results

### Proteogenomic coverage of breast cancer xenografts

**Figure 1:**
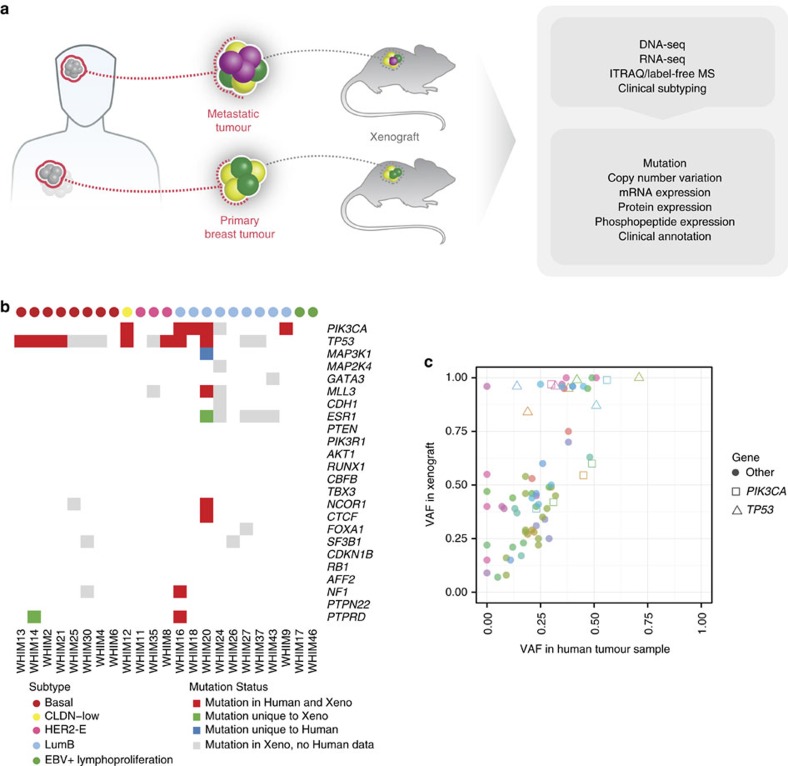
Modelling human breast cancer with patient-derived xenografts (*n*=24). (**a**) Illustration of generation and proteogenomic characterization of breast cancer xenograft models. (**b**) Somatic mutations of significantly mutated genes of human breast tumour were recapitulated in xenograft models. Mutation data for 23 WHIMs are shown (exome data were not available for WHIM47). (**c**) Variant allele fraction analysis showed clonal representation was consistent between human breast tumour and xenografts. Genomic driver events, including missenses and truncations in *TP53* and *PIK3CA*, were retained in the xenograft models. Each colour represents one xenograft sample.

We selected 24 PDX models established from primary or metastatic breast tumours for comprehensive proteogenomic characterization (Fig. 1a). The human patient cohort was composed of 10 basal, 1 claudin-low (CLDN-low), 9 luminal B and 4 HER2-enriched (HER2-E) breast tumours based on PAM50 expression subtyping (Supplementary Data 1). We conducted DNA and RNA sequencing respectively for 23 PDX models and in one case Sanger DNA sequencing of hotspot mutations. Isotope Tagging for Relative and Absolute Quantitation (iTRAQ 4-plex)^16^ was completed for all 24 PDXs for discovery and label-free quantification (LFQ) for 18 PDXs for validation and confirmation (Supplementary Data 2). The comprehensive proteogenomic coverage allowed us to systematically assess the somatic mutation profile, copy-number variation (CNV), mRNA expression, protein expression, and phosphosite levels and localizations.

We examined somatic mutations in significantly mutated genes of breast cancer in 12 human tumours and their matching PDXs (Supplementary Data 2). Most key somatic mutations in these genes were preserved (Fig. 1b), validating the genomic fidelity of these PDX models. However, recurrent breast cancer mutations were not detected in two PDX models (WHIM17 and WHIM46). While sequencing data were not available for the matched progenitor human tumours, the germline SNPs of human blood normal samples matched with the PDX tumours, validating their patients of origin. Follow-up histological and RNA-seq analyses suggested WHIM17 and WHIM46 are EBV-positive human lymphoproliferative cells arising in the NOD/SCID/γ mouse strain^17^ (Methods, Supplementary Fig. 1).

We then compared the variant allele fractions (VAFs) of exonic somatic mutations in human tumours and derived xenografts when both are available. We found comparable or higher VAFs in xenografts, potentially due to higher tumour purity and selection of some mutant alleles by loss of heterozygosity in PDX models (Fig. 1c). The relatively high, positive VAF correlation (*R*=0.66) implied similarity between human samples and their respective xenografts, consistent with our previous report^9^. Importantly, all *PIK3CA* and *TP53* somatic mutations in the 12 originating human tumours were detected in respective xenografts with comparable or increased VAFs (Fig. 1c). Among the 22 breast cancer PDX models, 14, including 7 of the 8 basal tumours, harboured *TP53* mutations, 5 luminal B PDXs had *ESR1* mutations, and 5 luminal B and 1 CLDN-low xenografts carried *PIK3CA* mutations.

We quantified mRNA expression of 16,209 unique human genes from RNA-seq data for these 24 models. We also applied two distinct MS data acquisition approaches (Supplementary Fig. 2a, Methods), iTRAQ (Supplementary Data 3) and LFQ (Supplementary Data 4), to quantify the expression ratios of 12,794 human proteins (11,879 genes) and 8,648 proteins (8,035 genes), respectively. A total of 56,874 phosphosites were also confidently identified using iTRAQ (Supplementary Data 5). After filtering for observation in at least 10 out of 24 samples (4 out of 9 of the iTRAQ experiments, Supplementary Fig. 3), the relative abundances of 10,069 proteins and 36,609 phosphorylation sites were quantified across tumours by iTRAQ and used in subsequent analyses in this study. The technical replicates in the LFQ (WHIM2 and WHIM16) and the iTRAQ (WHIM13) sets showed high correlation in protein expression levels (*R*>0.85, Supplementary Fig. 2b,c,e). Further, phosphosite expressions also showed high correlations in technical replicates (WHIM13) of the iTRAQ experiment (*R*=0.82, Supplementary Fig. 2d), validating the technical reproducibility of our proteomic and phosphoproteomic datasets. Of note, while iTRAQ and LFQ quantification were conducted separately and based on different features of the LC–MS data collected from the Q Exactive mass spectrometer (that is, reporter ion intensity and MS1 peak area, respectively), our analysis showed reasonable correlation between the two measurements after normalization (*R*=0.61). The two datasets utilizing the same PDX models but different workflows enabled cross-method validation of global proteomic results.

### Proteogenomic integration and comparison

**Figure 2:**
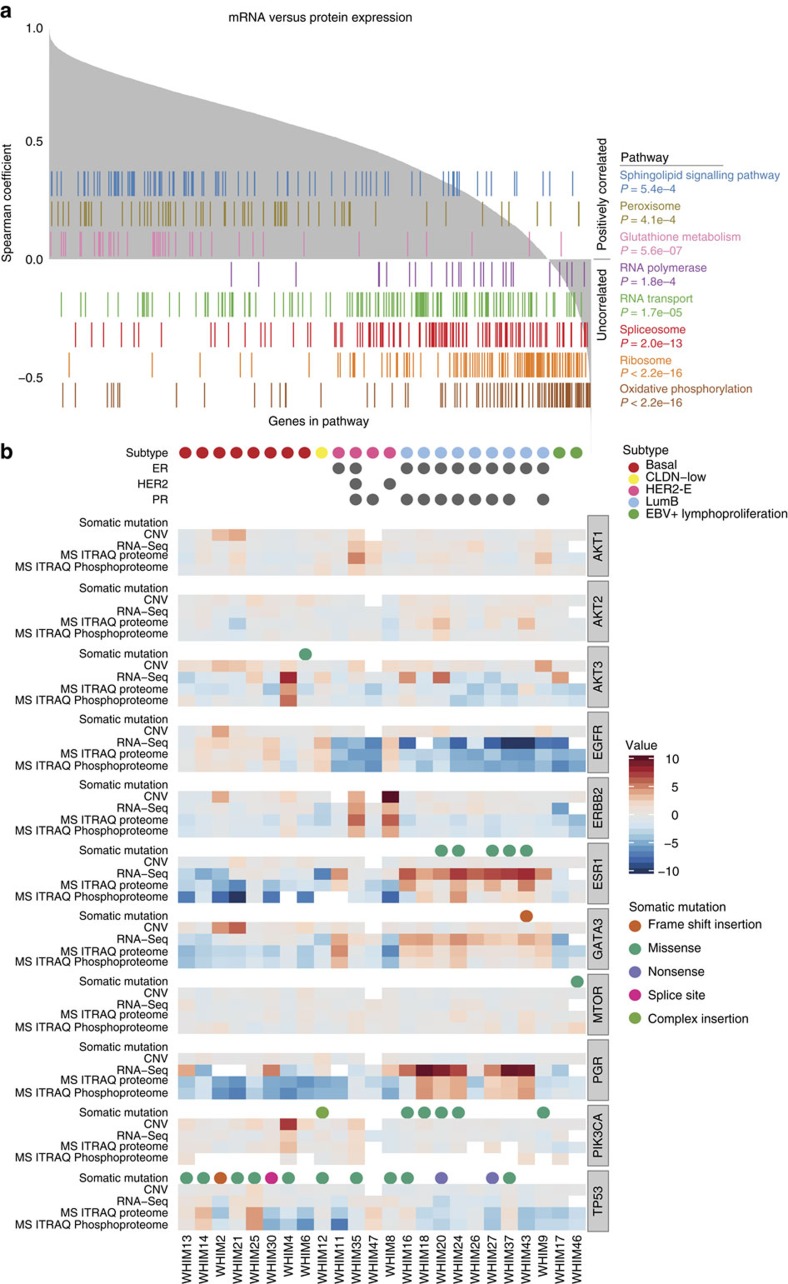
Proteogenomic correlation analysis in PDX samples. (**a**) Correlation between mRNA and iTRAQ protein expression levels identified pathways with significantly concordant or discordant mRNA-protein expressions. Genes were aligned along the *x* axis by the rank of their Spearman correlation coefficient between mRNA and protein expression levels. Each colour represents one significantly associated pathway, and each bar represents one gene in the pathway. (**b**) Proteogenomic summary of xenograft shows relationships among mutation, CNV (normalized log-R ratio), mRNA (log-transformed and normalized RSEM values), proteomic (normalized log2 ratio to reference), and phosphoproteomic expression (normalized log2 ratio to reference) levels of breast cancer-related genes in 24 PDX samples across 4 intrinsic subtypes. Expression values from each dataset were calculated as described (Methods) and truncated to a maximum of 10 and a minimum of −10 for visualization.

We first evaluated the correlation between mRNA expression and protein abundance measurements from the iTRAQ experiment (Fig. 2a) for these 24 PDX models: 83.6% of the genes with sufficient data showed positive correlations with a median Pearson *R*=0.536 (Supplementary Fig. 4). We investigated whether the trend of mRNA-protein correlations were associated with specific KEGG pathways^18^, finding that metabolic pathways involved in house-keeping functions are enriched for genes showing high, positive correlation. For example, genes in the glutathione metabolism pathway showed the highest enrichment for positive correlations (Kolmogorov–Smirnov test, false discovery rate (FDR)=5.6e-07). Interestingly, we observed that genes in the ribosome (FDR<2.2e-16), spliceosome (FDR=2.0e-13), RNA transport (FDR=1.7e-5), RNA polymerase (FDR=1.8e-4), and oxidative phosphorylation (FDR<2.2e-16) pathways showed relatively lower correlation between mRNA and protein level. These pathways were enriched for genes that do not require translated proteins for their biological functions. Similar pathway-specific pattern of positive and negative enrichment of correlations were observed by LFQ (Supplementary Fig. 5). The high degree of mRNA–protein correlation observed in the PDX samples is consistent with recent results obtained for human breast tumours^14^ and colorectal cancer^13^, suggesting that PDXs closely mimic the respective human breast tumours in their relationship between mRNA and protein.

We then examined the correlation of CNV, mRNA, and protein expression levels for several key genes for breast cancer biology: *EGFR*, *ERBB2* (*HER2*), *ESR1*, *GATA3*, *PGR*, *PIK3CA*, *AKT1/2/3*, *MTOR* and *TP53*. In most cases, we observed consistent relationships between CNV, mRNA, and protein expression levels. Compared with PDXs of non-luminal subtypes, luminal B breast cancer xenografts, as expected, showed higher mRNA and protein expression of *ESR1* and *PGR*, consistent with their positive ER and PR status (Fig. 2b). Five out of the six *PIK3CA* mutations were observed in luminal B PDXs, which tended to also show higher mRNA and protein expression levels of *GATA3*. In contrast, a larger proportion of basal PDXs expressed higher protein levels of EGFR. Strong HER2 expression at the mRNA, protein and phosphoprotein levels were detected in WHIM8 and WHIM35, both derived from HER2-positive breast cancers. Overall expression patterns for key genes were consistent with the clinical subtype diagnosis across CNV, mRNA, protein and phosphosite analyses (Supplementary Fig. 6).

### Proteomic subtyping of xenografts and human breast tumours

**Figure 3:**
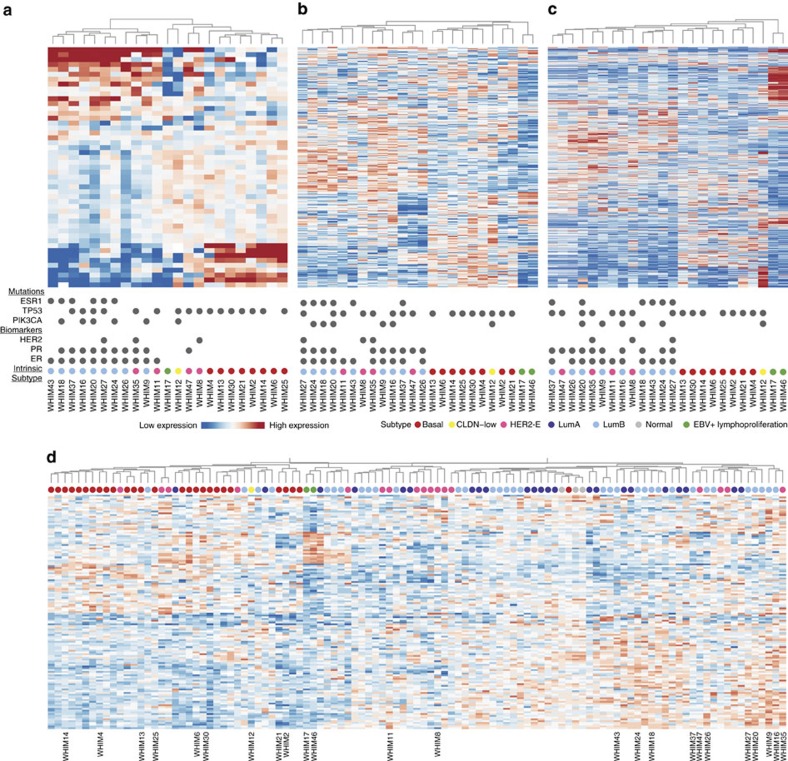
Transcriptomic and proteomic clustering of breast cancer PDX and human samples. (**a**) Transcriptomic clustering of PDX breast tumours based on the PAM50 gene expression markers. (**b**) Proteomic clustering of PDX breast tumours based on the top 436 variably expressed proteins. (**c**) Phosphoproteomic clustering of PDX breast tumours based on the top 1,737 variably expressed phosphosites. (**d**) Proteomic clustering using only 133 non-differential expressed proteins between WHIM and human breast tumour samples. The clustering reproduced the basal-enriched and luminal-enriched clusters, where PDX (*n*=24) and TCGA human breast tumour samples (*n*=77) cluster based on their subtypes. The non-differentially expressed proteins were identified through a *t*-test with FDR>0.3 between the PDX and the TCGA human tumour samples. The PDX tumours are labelled by their WHIM IDs whereas the human tumours are not labelled.

Molecular subtyping of breast cancer based on mRNA expression profiles has been shown to correlate with prognosis and has treatment implications^19^,^20^. As expected, transcriptome clustering based on PAM50 genes from RNA-seq data largely reproduced intrinsic subtypes of the breast tumours (Fig. 3a; Supplementary Data 6). To explore proteomic and phosphoproteomic subtype classifications in xenograft models, we conducted unsupervised clustering of proteomes and phosphoproteomes of the 24 xenograft samples based upon the top 436 variably expressed proteins showing a s.d. greater than 2 from the iTRAQ proteome (Supplementary Data 7). Two distinct clusters emerged: one contained all basal tumours and the only CLDN-low tumour (WHIM12), while the other included all luminal B and HER2-E breast tumours; HER2-E tumours did not show a proteomic expression profile distinct from luminal B samples (Fig. 3b). Clustering analysis using the same gene markers from the transcriptome (Supplementary Fig. 7a) and the LFQ proteome (Supplementary Fig. 7b; Supplementary Data 8) further supported the separation into these two proteomic subtypes, although the minor differences between the transcriptome and the proteome clustering suggested distinctions between mRNA and protein levels. The proteomic subtypes defined by the top 968 most variably expressed mouse host proteins did not segregate based on luminal and basal subtypes (Supplementary Fig. 8; Supplementary Data 9). Besides differing in mRNA expression, luminal and basal breast cancer PDXs also showed consistently distinct proteomic expression profiles, supporting their distinct biological origins.

We then utilized the iTRAQ phosphopeptide expression data to infer phosphoproteomic subtypes. 1,737 unique phosphosites with s.d. greater than 2.5 were included to conduct hierarchical clustering (Fig. 3c; Supplementary Data 10). These analyses of the phosphoproteomic data produced two major clusters segregating the luminal B and basal subtypes. Again, the WHIM37/WHIM47/WHIM26 group and the WHIM17/WHIM46 group, as observed in the proteome clustering, grouped closely together. Gene and protein expression of lymphoid lineage markers showed high expression of CD20 and JAK3 in WHIM17 and WHIM46 (Methods, Supplementary Fig. 9), consistent with their positive EBV status and histological diagnosis (Supplementary Fig. 1) as human lymphoproliferative cells arising in an immunocompromised mouse background. Overall, while the basal and luminal clusters remain consistent, the hierarchical distances between PDX samples within the two major clusters differ between data types. The departure of proteomic and phosphoproteome subtypes from mRNA expression-defined subtypes suggests independent layers of molecular heterogeneity provided by distinct proteomic analyses.

We then investigated whether human proteomic subtypes could be recapitulated in PDX models by including an additional 77 proteomes from TCGA human tumours that were processed concurrently in the iTRAQ experiment^14^ (Methods). To reduce clustering bias imbued by the mouse contribution to the proteome in the PDX samples, we excluded proteins showing differential expression between human tumour and xenograft (*t*-test, FDR≤0.3) (Supplementary Data 11). Additional requirements, including the presence of detectable protein in more than 10 samples and minimum difference of 2 s.d.'s in the merged proteome, resulted in 133 proteins qualifying for un-supervised clustering. Consistent with the subtyping analysis of the 24 PDXs alone, we identified two major clusters: one that included all but one basal breast tumour and the other comprised mostly of luminal tumours (Fig. 3d). Similar to the proteomic subtypes of the PDX cohorts, luminal tumours and HER-2 tumours did not show clear separation, although several sub-clusters were identified. Importantly, xenograft proteomes clustered adjacently to the human proteomes of their respective subtypes, validating the fidelity of basal and luminal proteomic signatures discovered in PDXs.

To search for defining markers between the basal and luminal B subtypes, we conducted differential expression analysis between the PDXs of the respective subtypes. We found several proteomic markers that were differentially expressed in both the LFQ and iTRAQ datasets (*t*-test, FDR≤0.05), including SPR, GSTP1 and SERPINB5 (Supplementary Fig. 10; Supplementary Data 12). We then conducted gene-set enrichment analysis based on the Reactome pathway database^21^ to investigate patterns of differential expression (Methods). The basal subtype breast tumours were up-regulated in most of the significantly differentially-expressed pathways (FDR≤0.01) identified by both LFQ and iTRAQ datasets, including extracellular matrix organization, cell cycle, and collagen formation. In comparison, the luminal B breast tumours showed higher expression in genes related to organelle biogenesis and membrane trafficking (Supplementary Data 13).

### Activated pathways revealed by phosphorylation profiles

**Figure 4:**
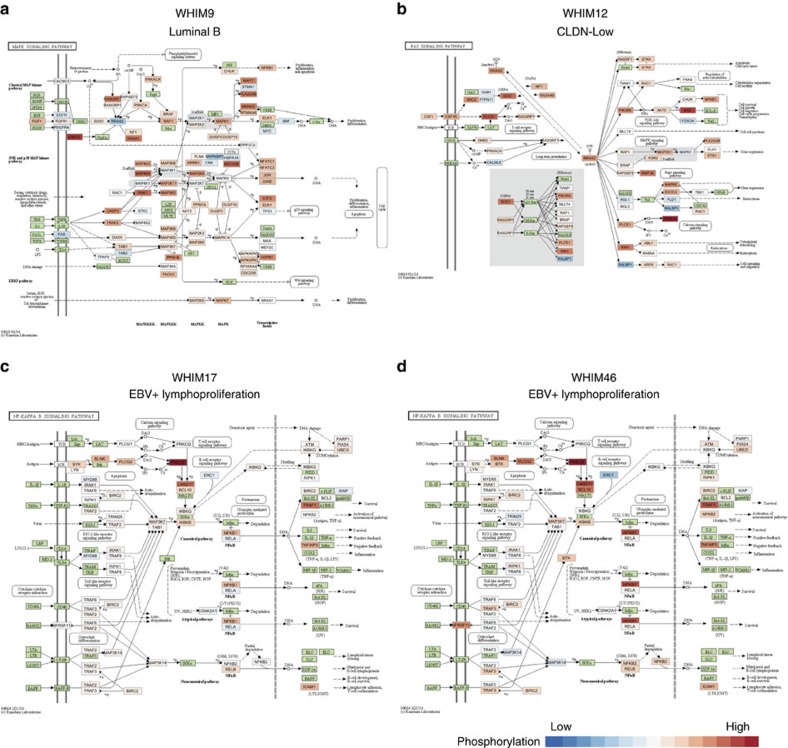
Activated signalling pathways detected through pathway phosphorylation enrichment analysis. (**a**) Activation of the MAPK signalling pathway in WHIM9. (**b**) Activation of the Ras signalling pathway in WHIM12. (**c**) Activation of the NFκB pathway in WHIM17. (**d**) Activation of the NFκB pathway in WHIM46. Phosphorylation levels of each protein in the pathway relative to the cohort of 24 PDX models are shown by the colour scale of red (high) to blue (low). Proteins with no phosphorylation data are coloured in green.

Cancer driving somatic events trigger major changes in downstream signalling to launch the tumorigenic cascade^22^. To search for tumour-specific activated pathways in PDX tumours, we systematically evaluated and compared phosphoproteome profiles of gene sets from KEGG signalling pathways (Methods). Phosphorylation enrichment analysis identified 12 significantly activated pathways, including Ras, MAPK and NFκB signalling, in 4 xenografts (FDR≤0.01, Supplementary Data 14). WHIM9 exhibited elevated phosphorylation of the MAPK signalling pathway (FDR=9.69e-6; Fig. 4a). Interestingly, WHIM9 carried a recurrent somatic mutation, *KRAS* p.A146V (refs 23, 24), which may have driven canonical MAPK pathway activation. Further, WHIM12 exhibited activation of the Ras signalling pathway (FDR=4.28e-5), along with an outlier protein expression of MET, a receptor tyrosine kinase upstream of the Ras signalling pathway (Fig. 4b). Interestingly, WHIM17 and WHIM46, both harboured BTK and PLCG2 protein overexpression, exhibited overall high phosphorylation of the NFκB signalling pathway (Fig. 4c,d; FDR=6.94e-3, 4.53e-5 respectively). This observation further supports the strong similarity between these two PDX models based on protein/phosphoprotein clustering (Fig. 3b,c) and their classification as EBV-positive lymphoproliferation.

While pathways such as PI3K/AKT/MTOR are known to be activated across the majority of breast tumours^22^, our analysis shows that other complementary tumorigenesis-related pathways including RAS/MAPK are also activated in a small set of breast tumours due to specific genomic or proteomic alterations, representing alternative treatment opportunities. In addition, our phospho-proteomic analysis revealed activation of signalling pathways not readily predicted by genomic data.

### Complementary genomic/proteomic druggable targets

We examined promising drug targets in each tumour by surveying the genome and expressed proteomes. Specifically, we compiled a list 76 druggable genes (Supplementary Data 15), along with their respective drugs, from established public databases (Methods). Six PDXs, representing 20.8% of the tumours in this study, harboured druggable somatic mutations, including *PIK3CA* p.H1047R and *KRAS* p.A146V in WHIM9, *PIK3CA* p.H1047R in WHIM16 and WHIM24, *PIK3CA* p.E545K in WHIM18, *PIK3CA* p.E542K in WHIM20 and *SF3B1* p.K700E in WHIM26 (Supplementary Data 16).

**Figure 5:**
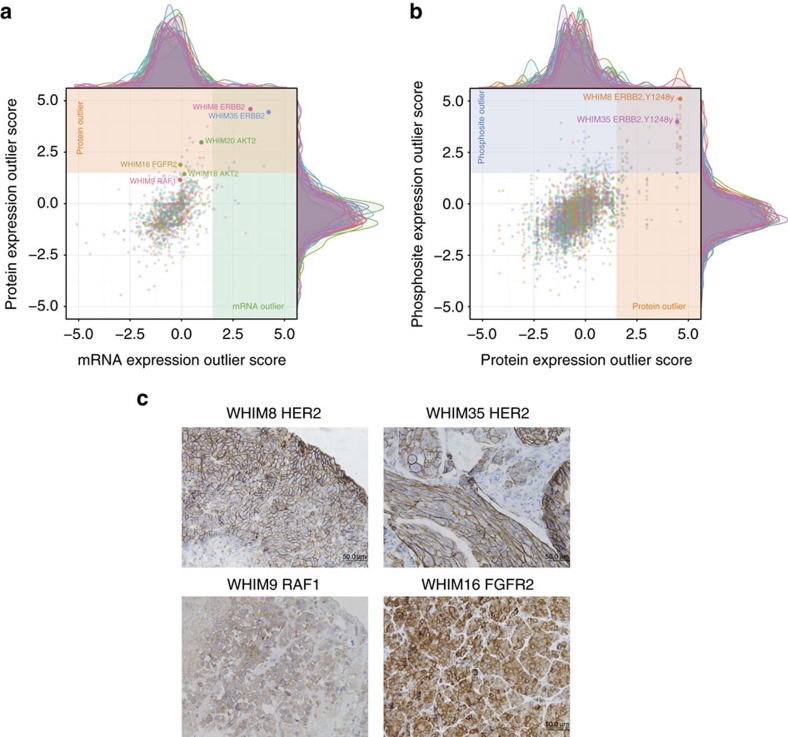
Druggable events identified by expression outlier analysis. Druggable outlier events identified at (**a**) the mRNA and protein and (**b**) protein and phosphopeptide expression levels. Each colour represents one xenograft sample. Key outlier events validated in this study are labelled by text. (**c**) Immunochemistry staining verified outlier expression of HER2 in WHIM8 and WHIM35, RAF1 in WHIM9, and FGFR2 in WHIM16. Scale bar: 50 μm.

While activating mutations in oncogenes can be targeted by treatments, aberrantly overexpressed or activated protein products, such as HER2, also presents exploitable treatment opportunities^25^,^26^,^27^. We sought genomic and proteomic evidence of overexpressed genes/proteins or proteins with highly phosphorylated sites. We defined outliers as expression values exceeding the 1.5 interquartile ranges (IQR) above the third quartile of the cohort (Variations of Box Plots), and rank ordered them by the outlier score. We further required CNV outliers to be validated with outlier expression in either the mRNA or protein level to rule out up-regulation events due to technical artifacts or passenger events (Methods). mRNA and protein expression outlier scores showed a moderate positive correlation (Fig. 5a, *R*=0.516), but mRNA outlier expression did not guarantee high protein expression (for example, AKT2 and FGFR2, Fig. 5a). Similarly, we observed a fraction of phosphosite outliers not detected at the protein level (Fig. 5b, *R*=0.548). Consequently, identifying post-transcriptional and post-translational events to capture potential druggable treatment opportunities requires consideration of protein expression as well as gene expression.

**Figure 6:**
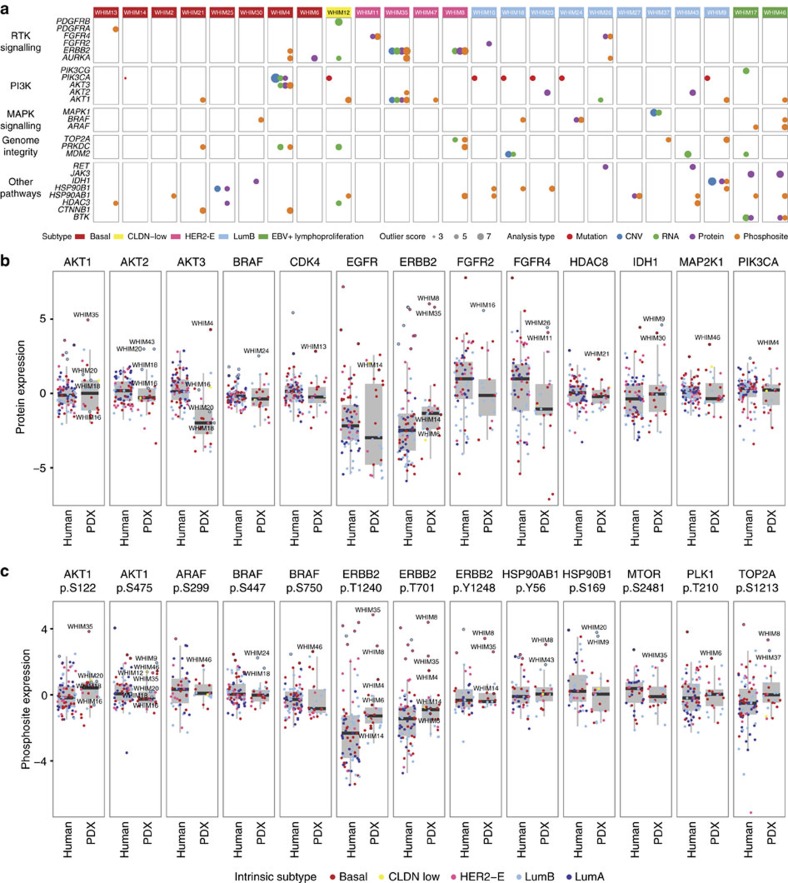
Druggable targets identified through proteogenomic analysis in PDX and human breast cancer. (**a**) Outlier analysis revealed potentially druggable events in the RTK, PI3K, MAPK signalling, genome integrity pathways at various frequency and magnitudes across four breast cancer subtypes. Selected genes with any outlier score greater than 2.5 or in the key oncogenic pathways, including the PI3K, RTK, MAPK signalling pathways, are shown. (**b**) Comparison of protein expression outliers of selected druggable genes in PDX and human breast tumours. (**c**) Comparison of overexpressed phosphosite outliers of selected druggable genes in PDX and human breast tumours. Key outlier events reaching the outlier definition threshold or validated in this study are labelled by text. For the box plots in (**b**) and (**c**), the center line indicates median of the protein/phosphosite expression in the human and PDX cohort. The upper and lower hinges correspond to the 25th and 75th percentiles; the upper whisker corresponds to 1.5 × IQR (inter-quartile range) above the 25 percentile and the lower whisker corresponds to 1.5 × IQR below the 75 percentile.

Applying this druggable outlier detection strategy across CNV, mRNA, protein and protein phosphorylation levels, we identified overexpressed druggable genes in 26.1% and 47.8% of PDXs at the CNV and mRNA levels, respectively (Fig. 6; Supplementary Data 16). These events recapitulated known druggable opportunities, such as the *PIK3CA* copy-number amplification in WHIM4 and *ERBB2* (*HER2*) copy-number amplification in WHIM35. Expanding to iTRAQ protein expression outliers allowed us to uncover druggable targets in 19 out of the 24 PDXs (79.2%), while considering phosphosite outliers covered 22 of the 24 PDXs (91.7%). A significantly high fraction of protein outliers overlapped between the LFQ and iTRAQ datasets (Fisher's Exact Test, *P*=2.013e-05, Supplementary Fig. 11), providing validation of our findings.

The identified proteomic outliers included multiple known druggable targets involved in the PI3K, RTK, MAPK signalling pathways and other oncogenic processes (Fig. 6; Supplementary Data 16). For instance, HER2 was found to be the top outlier at the protein expression level in 2 HER2-E xenografts, WHIM8 and WHIM35; the phosphosites of HER2 were also identified as outliers. Immunohistochemistry experiments validated the high HER2 protein expression on the cell membranes of WHIM8 and WHIM35 (Fig. 5c; Supplementary Fig. 12). Further, AKT1, AKT2 and AKT3 proteins were outlier expression candidates in WHIM35, WHIM20 and WHIM43 and WHIM4, respectively, providing proteomic validation of previously observed AKT up-regulation at mRNA expression level in breast cancer^22^. Other outlier proteins included IDH1 in WHIM9 and WHIM30, FGFR4 in WHIM11 and WHIM26, and both BTK and JAK3 in the EBV-positive WHIM17 and WHIM46. Many of these protein outliers aligned with up-regulation in their corresponding phosphosites, including phosphosites on AKT1, AKT3, BRAF, FGFR4 and HSP90AB1 (Fig. 5c, selected phosphosite spectra in Supplementary Fig. 13). We also discovered additional outlier phosphosites in other proteins including CTNNB1, ARAF, and HSP90B1 (Supplementary Data 16). Finally, we identified outlier FGFR2 in WHIM16 and high RAF1 in WHIM9 and further validated their high protein expression status of by immunohistochemistry analyses (Fig. 5c; Supplementary Fig. 12). As diverse sets of proteomic outliers are identified across PDXs, effectively inhibiting such activation events will be required when designing targeted treatment strategies to each individual breast tumour.

Notably, for 80.6% of proteomic outlier events, we were able to identify human tumours from the 77 TCGA samples^14^ showing the same outlier protein expression through outlier score or ranking (Methods, Fig. 6b, Supplementary Data 16). For example, we observed outlier HER2 expression in five HER2-E and four luminal B human breast tumours. Further, a basal human sample carried both the outlier FGFR2 and FGFR4 expression, validating the findings in WHIM16, WHIM26 and WHIM11. Basal human breast tumours also carried protein expression outliers in IDH1, EGFR and MAP2K1 (Fig. 6b). Phosphosite outlier expression events showed a moderate rate of validation in the same human cohort (48.8%, Fig. 6c), suggesting its transient nature and potential micro-environmental effects on protein phosphorylation. As expected, HER2-E and a few HER2-positive luminal B tumours carried outliers in HER2 phosphosites including p.T701, p.T1240 and p.Y1248. ARAF phosphosite outliers, such as p.S299, were found in both human and PDX samples, validating our previous finding in the human cohort^12^. Interestingly, we identified outlier phosphosites in genes not previously implicated in breast cancer through genomic profiles, such as BRAF p.S447, p.S750 and HSP90AB1 p.Y56 and p.S169 (Fig. 6c). Our results demonstrated that proteomic outlier events, like genomic driver mutations, are consistently observed in PDXs and human tumours. Some protein outlier events might represent ‘proteomic drivers' of tumorigenesis and therefore potential drug targets in breast tumours.

### Targeted treatments using breast cancer xenograft models

**Figure 7:**
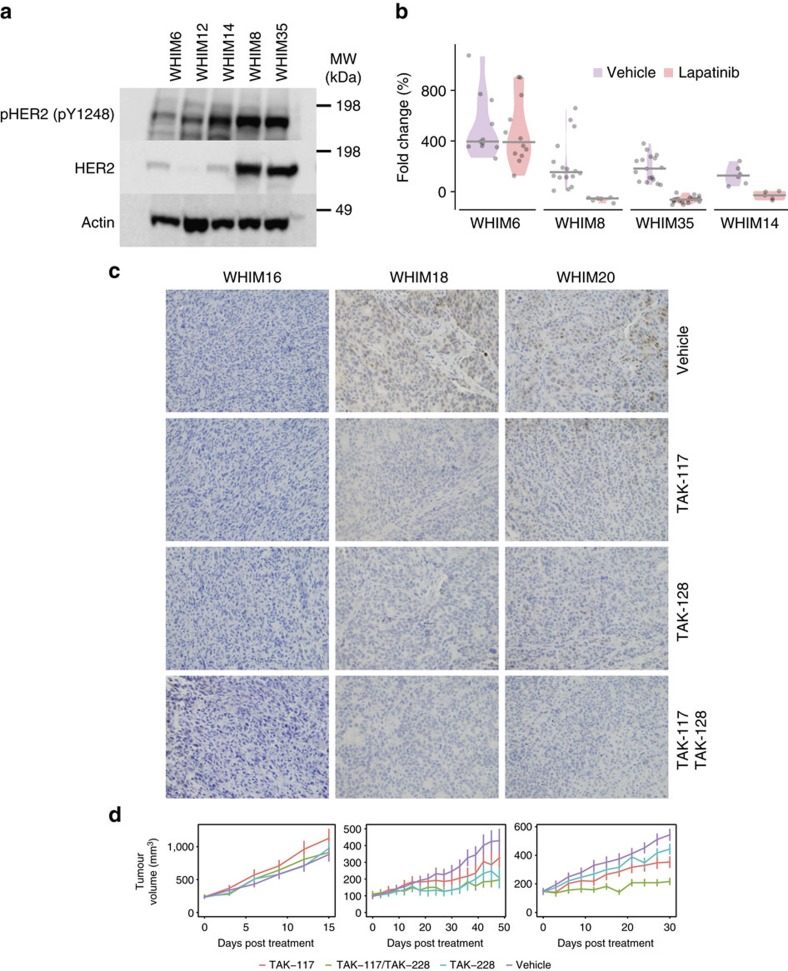
Targeted treatments of breast cancer xenografts. (**a**) Western blot of HER2 protein and HER2 p.Y1248 expression levels in 5 WHIM models (WHIM6, WHIM8, WHIM12, WHIM14 and WHIM35). (**b**) *In vivo* treatment responses to lapatinib in 4 PDX models including two HER2 positive lines (WHIM8 and WHIM35) and two basal lines (WHIM6 and WHIM14). The response is measured in fold change (%) of tumour volumes after 2 weeks of vehicle or lapatinib treatment. (**c**) Immunochemistry staining of AKT phosphorylation status in WHIM16, WHIM18 and WHIM20 in response to PI3K inhibitor TAK-117, mTOR inhibitor TAK128, and TAK-117/TAK-128 combined. (**d**) *In vivo* treatment responses to PI3K/mTOR inhibitors (TAK-117/TAK-128) of WHIM16, WHIM18 and WHIM20 (*n*=8-9 for each control or experimental group). Values were represented by average tumour volume (mm^3^) every 3 days following treatment (error bars: s.e.m.).

To validate the identified druggable events, we conducted treatment experiments targeting HER2 and PI3K pathways on selected PDX models. Four PDX models were chosen to address HER2 targeting using lapatinib, an oral HER2 kinase inhibitor. These include 2 HER2-E PDX models WHIM8 and WHIM35, both with high HER2 protein and phosphoprotein expression, and 2 low HER2 expressing, basal-like PDX models WHIM6 and WHIM14. Western blotting suggested high HER2 expression and phosphorylation of HER2 p.Y1248 in both HER2 positive tumours, WHIM8 and WHIM35 (Fig. 7a; Supplementary Fig. 14). Unexpectedly, high levels of HER2 p.Y1248 phosphorylation was also detected in WHIM14 by western blotting. In contrast MS-based phosphoproteomics also detected high levels of HER2p.Y1248 in WHIM8 and WHIM35, but not in WHIM14, which was more consistent with the known biology of the models. Antibody-based diagnosis of HER2 activation in WHIM14 was possibly due to cross-reaction of the Y1248 antibody with the pY1172 site of EGFR that bears high sequence similarity around the pY residue (..GTPTAENPEy_1248_LGLDVPV-CO_2_H vs...GSTAENAEy_1172_LRVAPQ.., Supplementary Fig. 6). As expected, WHIM8 and WHIM35 were growth inhibited (Wilcoxon Rank Sum Test, *P*=2.7e-5, 4.8e-7) by lapatinib, whereas WHIM6 was not (*P*=0.65) (Fig. 7b). Interestingly, WHIM14 also showed significant reduction in tumour growth by lapatinib (*P*=4.3e-3). Upon further exploration with a lower, clinically achievable dose of lapatinib for chronic treatment (30 mg kg^−1^ compared with 220 mg kg^−1^ in the previous experiment) over 48 days, significant but weak tumour growth inhibition was again achieved (*P*=0.0311, Supplementary Fig. 15). However, WHIM14 did not respond to HER2/HER3 antibodies trastuzumab or pertuzumab (*P*=0.250 and 0.181, respectively; Supplementary Fig. 15). Thus, the response of WHIM14 to lapatinib was likely due to inhibition of EGFR not HER2 (refs 28, 29). While WHIM6 also showed elevated EGFR protein level, WHIM14 showed notably higher EGFR phosphorylation based on both mass spectrometry (Fig. 2b) and western blotting (Fig. 7a), which could account for their different response to lapatinib. Two basal and one HER2-E human breast cancers harboured outlier EGFR expression (Fig. 6b), suggesting EGFR remains a potential therapeutic target in a subset of breast cancers that has yet to be fully realized clinically.

The PI3K/AKT/mTOR signalling pathway is altered in approximately 77% of breast tumours^22^ and multiple drugs targeting its components, including Class I PI3Ks, AKTs, and mTORs, are already in clinical trials^30^. Among these, a combination of everolimus and exemestane has been approved for treating advanced ER-positive breast cancers resistant to non-steroidal aromatase inhibitors^31^. Promising activity has also been reported for direct inhibitors of PI3K^32^,^33^. However, mutations in PIK3CA and other genetic alterations at the genomic level have so far failed to closely predict therapeutic responsiveness to PI3K pathway inhibitors^32^. We therefore hypothesized that combined genomic and proteomic indication of PI3K signalling activation is necessary for the prediction of treatment response. Our integrated approach identified 6 xenografts that harboured complementary genomic and proteomic druggable events in the PI3K-AKT pathway. In particular, WHIM16 harboured a hotspot *PIK3CA* p.H1047R mutation, whereas WHIM18 and WHIM20 each carried a hotspot *PIK3CA* p.E545K mutation. WHIM20 also showed an additional outlier protein expression of AKT2 and WHIM18 also expressed AKT2 at a near outlier level that may combine to activate PI3K pathway signalling (Fig. 6). Since the treatments were applied to later-passage PDX models relative to the ones we conducted proteogenomic analysis on, we performed immunohistochemistry to validate the phosphorylation of AKT p.S473. WHIM18 and WHIM20 showed detectable AKT p.S473, which was not observed in WHIM16 (Fig. 7c).

We conducted combinatorial treatment experiments by applying an alpha specific PI3K inhibitor (TAK-117) and/or an mTORC1/2 inhibitor (TAK-228) to three PDX models of luminal B breast cancer. Consistent with previous reports, *PIK3CA* mutation status alone did not accurately predict outcome; WHIM18 and WHIM20 showed reduced tumour growth upon application of the inhibitors, whereas WHIM16 did not (Fig. 7d). mTOR inhibition repressed tumour growth in WHIM18 and WHIM20 (ANOVA followed by Tukey's *post hoc* test, *P*=9.5e-10, 1.6e-03 respectively), but not in WHIM16 (*P*=0.97), showing that inhibition of mTORC1/2 may effectively suppress breast tumours with activated AKTs and validating a previous study showing the efficacy of mTOR inhibition in PDX models of triple-negative breast cancer^34^. Importantly, the combinatorial treatment achieved the greatest effect in WHIM18 and WHIM20 (*P*<2.2e-16 for both comparisons). While neither PI3K nor mTOR inhibitor drug treatment alone suppressed tumour growth in WHIM20 completely, the combination of both PI3K and mTOR inhibitors significantly reduced tumour growth to a nearly static state (Fig. 7d). Based on our proteomic characterization, WHIM20 exhibited the strongest AKT1 and AKT2 protein expression, as well as AKT1 p.S122 and p.S475 phosphorylation signatures followed by WHIM18 and then WHIM16 (Fig. 6b,c). Further, both PI3K and mTOR inhibitors significantly reduced AKT p.S473 (Fig. 7c). Our treatment results showed that the magnitude of drug response may be associated with overexpression and phosphorylation of the downstream signalling targets such as AKT proteins.

In addition to this validation of druggable hypotheses in luminal tumours, our previous report also demonstrated the effectiveness of combinatorial therapy of AKT and mTOR inhibitors in two other basal breast cancer xenografts^35^. One of the treated xenografts, WHIM4, was also characterized in this study, and showed copy-number amplification of *PIK3CA* and AKT3 protein outlier expression (Fig. 6). Overall, proteogenomic analysis revealed that the dual activation of PIK3CA at the genomic level and AKTs at the protein level may be a common signature of breast tumours, affecting more than 20% of PDXs in this cohort. Importantly, our results demonstrate the potential utility of combinatorial inhibitor treatments to treat breast tumours showing these proteogenomic signatures.

## Discussion

Breast cancer has been traditionally characterized in the clinic through hormone receptor status and selected genes' expressions^20^,^36^, and more recently by genomic sequencing^3^. However, druggable genomic driver events are detectable in only a limited percentage of patients^3^. As the majority of drugs target proteins, a systematic evaluation of breast cancer proteomes would seem ultimately to be necessary for selecting targeted treatment and predicting drug response. Recent advances in MS-based proteomics allow extensive and quantitative surveys of the global proteome. Here, we have systematically analyzed proteogenomic profiles of 22 patient-derived breast cancer xenografts and 2 EBV-positive lymphoproliferations that are likely artifacts of engraftment of human lymphocytes into NSG mice.

This study shows that proteogenomic signatures of PDXs resemble most findings from breast cancer patients. While some discrepancies exist, we established a normalization strategy at both the genomic and proteomic levels that enabled direct comparison. PDX tumours recapitulated the proteomic diversity of human breast cancers (Fig. 3). We also identified multiple druggable targets for each tumour model (Fig. 6; Supplementary Data 17). Proteomic events validated a significant number of CNV or mRNA up-regulations. For example, HER2 protein and phosphosite outlier expression was observed in HER2-E WHIM8 and WHIM35 (Fig. 5), which were effectively treated using lapatinib (Fig. 7b). Interestingly, we also identified overexpressed proteomic events not evident in the genomic level in both PDX and human samples, including outlier protein expression of EGFR, and outlier phosphosite expressions of ARAF, BRAF, HSP90AB1, PTPN11 and TOP2A (Fig. 6), highlighting potential new treatment opportunities in breast cancer. In the two PDX models subsequently diagnosed as EBV-positive lymphoproliferations, we observed outlier BTK expression (Fig. 6) and activation of the NFκB pathway (Fig. 4), validating BTK as a druggable target in EBV+ lymphomas^37^. While more than 80% of the proteomic outlier events in PDX were also found in human tumours, a lessor 48.8% of phosphosite outlier events were validated, potentially due to different tumour micro-environments. Thus, transient phosphoproteomic events identified in PDX tumours would likely require further verification in their corresponding primary tumours.

Outlier protein expression events can likely lead to downstream pathway activation, such as MET outlier protein expression (Fig. 6) and activated Ras pathway observed in WHIM12 (Fig. 4). While genomic analysis has utilized mutual occurrence or exclusivity in patient cohorts to deduce pathway relationships, phosphorylation profile analyses allowed us to directly interrogate signalling in these established pathways in a single sample. Our pathway activation results suggest these events may be crucial to tumorigenesis and that some are likely the proteomic ‘smoking guns' that originally triggered the oncogeneic cascade.

Roughly 77% of breast tumours showed alterations in the PI3K-AKT signalling pathway, representing a potentially important path forward for drug-based treatment. Yet, genomic alterations of PI3K pathway components have not shown themselves to predict treatment responsiveness to PI3K pathway inhibitors^32^. In this study, we defined complementary druggable targets of the pathway using proteogenomic analysis, including several events of a co-occurring PIK3CA mutation or copy-number amplification, and AKT protein outlier expression coupled by elevated AKT phosphorylation. In two such breast tumours, we successfully inhibited tumour growth using a combination of PI3K and MTOR inhibitors (Fig. 7c). While these results show potential functional implications, additional, systematic treatment experiments are required to validate the identified proteomic druggable targets.

In conclusion, this initial work using proteogenomic integration coupled with patient-derived xenograft validation, has demonstrated a strategy that, in principle, may enable more accurate prediction of the efficacy of mechanism-based cancer therapeutics.

## Methods

### Xenograft model generation

Patient-derived xenografts were generated from primary or metastatic breast tumours using previously described procedures^9^. All human tissues for these experiments were processed in compliance with NIH regulations and institutional guidelines, and approved by the institutional review board at Washington University. All animal procedures were reviewed and approved by the institutional animal care and use committee at Washington University in St. Louis. PDX models are available through the application to the Human and Mouse-Linked Evaluation of Tumors core at http://digitalcommons.wustl.edu/hamlet/.

We selected 24 of the established breast tumour samples, including 9 luminal B, 10 basal, 4 HER2-E, and 1 CLDN-low breast tumours for further proteogenomic characterization. Receptor statuses of xenograft tumours were validated using IHC after engraftment.

### Immunohistochemistry

Xenografts were formalin-fixed at least for 24 h and paraffin-embedded. Sections were evaluated by hematoxylin & eosin (H&E) staining. Immunohistochemistry (IHC) was performed on additional sections for HER2 (Dako), Phospho-Akt (Ser473) (Cell signalling), FGFR2 (Abcam), and Raf-1 (Santa Cruz) following the manufacturer's instructions. Western blotting of phosphorylated HER2 (p.Y1248) was performed using antibody cat Nr-06-229 (Millipore).

### *In vivo* drug treatment experiments

For the targeted treatment of the PI3K pathway, PI3K alpha inhibitor TAK-117 and TORC1/2 inhibitor TAK-228 were provided by Millennium Pharmaceuticals, Inc. The compounds were dissolved in Peg400. Tumours were engrafted in NSG (NOD.Cg-*Prkdc*^*scid*^ *Il2rg*^*tm1Wjl*^*/*SzJ) mice (The Jackson Laboratory) by subcutaneous injection of 2–5 × 10^6^ PDX cells in PBS supplemented with 30% Matrigel (BD Biosciences, cat. no. 354234). When tumours reached an average size of 250–300 mm^3^, animals were assigned randomly to control and various treatment groups (*n*=8–9 each group). For treatment of WHIM16 (passage 7), 18 (passage 8), and 20 (passage 5) were used. Tumour bearing mice were gavaged with: (1) Peg400; (2) TAK-117, 140 mg kg^−1^ day^−1^; (3) TAK-228, 1 mg kg^−1^ day^−1^; (4) TAK-117, 140 mg kg^−1^ day^−1^, TAK-228, 1 mg kg^−1^ day^−1^. The mice were treated on three consecutive days once daily and then had a 4-day interval. Tumours were measured with external caliper, and volume was calculated as (4*π*/3) × (width/2)^2^ × (length/2).

For the lapatinib therapeutic experiments of WHIM6 (passage 7), WHIM 14 (passage 11), WHIM 8 (passage 6) and WHIM 35 (passage 6), 1 × 10^6^ tumour cells were added to equal volume of 1:1 mixture of Matrigel and 10% RPMI plus 10% FBS to 4th mammary fat pads in female SCID/bg mice (ENVIGO). We then established tumours to an average volume 250–300 mm^3^, and randomized the mice into control and lapatinib groups. We treated the treated group mice with lapatinib chow diet by formulating lapatinib (220 mg kg^−1^) and processing them into food pellets (by Research Diets Inc), which is supplied for 2 weeks. The numbers of replicates for each group are as followed: WHIM 14 control (*n*=6) and lapatinib treated (*n*=5); WHIM 6 control (*n*=11) and lapatinib treated (*n*=12); WHIM 8 control (*n*=16) and lapatinib treated (*n*=6); WHIM 35 control (*n*=17) and lapatinib treated (*n*=18). We also treated WHIM14 with 100 mg kg^−1^ lapatinib treatment for 48 days, including both control (*n*=8) and lapatinib treated (*n*=9) groups. Further, we tested the effect of trastuzumab (30 mg kg^−1^ weekly with IP injections) and pertuzumab (30 mg kg^−1^ weekly with IP injections) on PDX tumour growth for 48 days. The experimental groups are as following: control group (*n*=6) treated with physiological saline (vehicle); trastuzumab treated group (*n*=12); pertuzumab treated group (*n*=11).

Statistical testing of the resulting data was conducted using the R programming language. Wilcoxon rank sum test was applied to compare the fold change in tumour volumes after two weeks in the lapatinib treatment experiment, and after 44 days or 48 days for the additional WHIM14 experiment using low-dose lapatinib and trastuzumab/pertuzumab. One-way ANOVA was applied to compare the treated versus control groups in the PI3K targeted-therapy experiments, and a follow-up Tukey's *post hoc* test was used for the various comparisons in PI3K inhibition experiments. All human tissues for these experiments were processed in compliance with NIH regulations and institutional guidelines, and approved by the Washington University, University of North Carolina, or Baylor College of Medicine Institutional Review Board (IRB). All animal procedures were reviewed and approved by the institutional animal care and use committee at Washington University in St. Louis, University of North Carolina, or Baylor College of Medicine.

### Genomic and proteomic data generation.

#### Somatic mutation

Sequencing reads were aligned using BWA^38^. Somatic variants were identified using VarScan2 (refs 39, 40, 41), GATK^42^ and Pindel^43^, and annotated based on Ensembl release 70_37. We then filtered out common variants by using variants from the 1000 Genomes and NHLBI projects. We further eliminated mouse contamination by filtering somatic variants that were mapped to mouse reference genome. Somatic mutation calls were validated either using a custom array or manually reviewed in IGV. The genomic data of 17 out of the 24 PDXs have been presented before in our previous studies^6^,^8^ (Supplementary Data 2).

#### Copy-number variation

The segment-based copy-number data were generated using the whole-genome sequencing and exome sequencing data. We then converted the segment-based copy-number data to the gene-based copy-number data by using the RefSeq database (version 20130727). The copy-number values were further transformed to the log-R ratio, using the cohort mean for the gene as the reference.

#### mRNA expression and virus detection through RNA-seq

mRNA expression values were calculated from mRNA sequencing data using MapSplice^44^,^45^. The resulting RSEM values were normalized within samples to a fixed upper quartile. Upper quartile normalized RSEM data were log2 transformed and the data were median centred by gene. To quantify virus abundance, we used the VirusScan pipeline (https://github.com/ding-lab/VirusScan) to detect viruses by numbers of virus-supporting reads from RNA-seq data.

#### LFQ proteome

Tumour Sample Generation and Protein Extraction. Patient-derived xenograft breast tumours were processed to cryopulverized powders as described previously^46^. The powders (100 mg wet weight) were subjected to lysis and protein extraction using a buffer composed of 8 M urea, 50 mM Tris pH 8.0, 75 mM NaCl, 1 mM MgCl_2_, and 500 units Benzonase. Approximately 1 mg of total protein extracted was reduced with DTT and subsequently alkylated with iodoacetamide. The proteins were then subjected to proteolysis with endoproteinase Lys-C (Wako Chemicals, USA) for ∼4 h at 37 °C. The solution was diluted 4-fold with 25 mM Tris pH 8.0, 1 mM CaCl_2_ and further digested with trypsin (Promega) for ∼12 h at 37 °C. Digestion was stopped by the addition of TFA to 0.4%, and the precipitate was removed by centrifugation. The peptide solutions were desalted on Sep-Pak Light C18 cartridges (Waters) and dissolved in 30% ACN, 0.1% TFA before loading on a 300 μm Source 15 S (GE Healthcare) column for Basic Reversed Phase Chromatography (bRPLC)^47^. A linear LC gradient was performed by increasing buffer B from 0 to 70% within 60 min, where buffer A was aqueous 10 mM ammonium formate, and buffer B was 90% ACN in 10 mM ammonium formate. A total of 30 fractions were collected for each WHIM sample (18 WHIMs). Five fractions were then prepared by combining non-contiguously fractions. We analyzed an additional technical replicate for WHIM2 and WHIM16. The fractions were dried and desalted using a stop-and-go-extraction tip (StageTip) protocol containing 4 × 1 mm C18 extraction disk (3M).

Liquid Chromatography-Tandem Mass Spectrometry and Protein Identification. Sample analysis was performed via reversed phase LC-MS/MS using a Proxeon 1000 nano LC system coupled to a Q Exactive mass spectrometer (Thermo Scientific, San Jose, CA). The Proxeon system was configured to trap peptides using a C18 column (3 cm × 100 μm i.d.) with a diverted flow rate (5 μl min^−1^) The trap column was placed in line with the analytical column (15 cm × 75 μm i.d., 3.5 μm, 300 Å particle C18, Thermo Scientific, San Jose, CA, USA) before gradient elution of peptides. Analytical separation of all the tryptic peptides was achieved with a linear gradient of 2–30% buffer B over 240 min (250 nl min^−1^), where buffer A was aqueous 0.1% formic acid, and buffer B was acetonitrile in 0.1% formic acid.

LC-MS experiments were performed in a data-dependent mode with Full-MS (externally calibrated to a mass accuracy of <5 p.p.m., and a resolution of 70,000 at *m/z*=200) followed by HCD-MS/MS of the top 20 most intense ions. High-energy collision activated dissociation (HCD)-MS/MS was used to fragment peptides at a normalized collision energy of 27 eV in the presence of nitrogen. One LC-MS run was performed for each fraction (from 1 process technical replicate), except for WHIM2 and WHIM16 where 2 LC–MS runs were conducted (from 3 process technical replicates), resulting in the production of 100 LC–MS runs for global peptide analysis. Mass spectra were processed, and peptide identification was performed using the Andromeda search engine found in MaxQuant software ver. 1.5.0.25. (Max Planck Institute, Germany). All protein database searches were performed against the RefSeq database (version 20140707). Peptides were identified with a target-decoy approach using a combined database consisting of reverse protein sequences of the RefSeq human, mouse and common repository of adventitious proteins (cRAP). The cRAP database was obtained from the Global Proteome Machine (ftp://ftp.thegpm.org/fasta/cRAP). Peptide inference was made with a FDR of 1% while peptides were assigned to proteins with a protein FDR of 5%. A precursor ion mass tolerance of 20 ppm was used for the first search that allowed for *m/z* retention time recalibration of precursor ions that were then subjected to a main search using a precursor ion mass tolerance of 6 p.p.m. and a product ion mass tolerance 0.5 Da. Search parameters included up to two missed cleavages at KR on the sequence, and oxidation of methionine, and protein N terminus acetylation as a dynamic modification. Carbamidomethylation of cysteine residues was considered as a static modification. Peptide identifications are reported by filtering of reverse and contaminant entries and assigning to their leading razor protein according to the Occams razor principal. The mass spectrometric data are deposited at the CPTAC Data Coordinating Center as raw and mzML files (https://cptac-data-portal.georgetown.edu)^47^.

Peptide and Protein Quantitation. LFQ was performed based on peak areas. The measured area under the curve of *m/z* and the retention time-aligned extracted ion chromatograms of peptides were performed via the label-free quantitation module in MaxQuant [ver. 1.5.0.25]^48^. All replicates for each PDX were included in the LFQ experimental design with peptide-level quantitation performed using unique and razor peptide features corresponding to identifications filtered with a posterior error probability (PEP) of 0.01, peptide FDR of 0.01 and protein FDR of 0.05. The expression values were median centred in the Perseus software for further analysis [version 1.5.0.9].

#### iTRAQ proteome and phosphoproteome

We included all 24 of the established breast tumour samples for proteomic characterization using iTRAQ. Tumour tissue samples were maintained in cryovials at −80 °C until cryopulverization using a CP02 Cryprep Pulverizer (Covaris, Woburn, MA). 90 mg aliquots of cryofractured material were prepared for proteomic processing in aluminum weighing dishes on dry ice using spatulas kept cold in liquid nitrogen, with remaining material reserved for other applications. The 90 mg target was designed to include 40 mg for each of the collaborating research teams, with an anticipated yield for each team of 1.5–2 mg protein based on 4–5% recovery. To avoid systematic bias, sample processing was block randomized, with each intrinsic subtype proportionally represented in each processing tranche.

The reproducibility of the iTRAQ4-plex global proteome and phosphoproteome analysis workflow used in this study has been extensively tested for quantitative reproducibility both within and across laboratories in the CPTAC program^14^,^49^. Over a period of several months 5 iTRAQ4-plex replicates were measured at each of the 3 CPTAC proteome analysis centres. Each of these iTRAQ4-plexes contained duplicate measurements for both a basal WHIM2 and a luminal WHIM16 PDX samples that are also part of this study. A high degree of consistency in the number of proteins identified and correlation in the protein expression was obtained^49^. Pearson correlations for replicate proteome and phosphoproteome measurements were very high with a *R*=0.9 in our previous study^14^ and very similar to the correlation observed here for the WHIM13 replicate measurement. These data show that our platform provides highly reproducible quantitative measurements for global proteomes and phosphoproteomes.

Protein extraction, digestion and iTRAQ labelling of peptides from breast cancer tumours. Cryopulverized breast cancer tumour samples tissues (∼2 combined aliquots of 90 mg tissue weight each) were homogenized in 1,000 μl lysis buffer containing 8M urea, 75 mM NaCl, 1 mM EDTA in 50 mM Tris HCl (pH 8), 10 mM NaF, phosphatase inhibitor cocktail 2 (1:100; Sigma, P5726) and cocktail 3 (1:100; Sigma, P0044), 2 μg ml^−1^ aprotinin (Sigma, A6103), 10 μg ml^−1^ Leupeptin (Roche, #11017101001), and 1 mM PMSF (Sigma, 78830). Lysates were centrifuged at 20,000 *g* for 10 min before measuring protein concentration of the clarified lysates by BCA assay (Pierce). Protein lysates were subsequently reduced with 5 mM dithiothreitol (Thermo Scientific, 20291) for 45 min at room temperature, and alkylated with 10 mM iodoacetamide (Sigma, A3221) for 45 min in the dark. Samples were diluted 4-fold with 50 mM Tris HCl (pH 8) prior to digesting them with LysC (Wako, 129-02541) for 4 h and trypsin (Promega, V511X) overnight at a 1:50 enzyme-to-protein ratio at room temperature overnight on a shaker.

Digested samples were acidified with formic acid (FA; Fluka, 56302) to a final volumetric concentration of 1% or final pH of ∼3–5, and centrifuged at 2,000 g for 5 min to clear precipitated urea from peptide lysates. Samples were desalted on C18 SepPak columns (Waters, 100 mg, WAT036820) and 1 mg peptide aliquots were dried down using a SpeedVac apparatus.

Construction of the Common internal Reference Pool. The proteomic and phosphoproteomic analyses of xenograft samples were performed as iTRAQ 4-plex experiments. Quantitative comparison between all samples analyzed was facilitated by the use of iTRAQ reporter ion ratios between each individual sample and a common internal reference sample present in each 4-plex. The reference sample was comprised of 16 of the 24 WHIM tumours analyzed in this study with equal contribution for each tumour (WHIM numbers 2, 4, 6, 8, 11, 12, 13, 14, 16, 18, 20, 21, 24, 25, 30 and 46). The 24 tumour samples were analyzed in nine independent 4-plex experiments, with three individual samples occupying the first three channels of each experiment and the 4th channel being reserved for the reference sample. While eight iTRAQ 4-plex experiments were used to analyze the 24 individual WHIM tumour samples, an additional 4-plex experiment was designed to include the WHIM13 sample for process replicate analysis and also internal reference samples from our human primary breast cancer study^14^ and a taxol drug response study (unpublished) to allow cross-referencing of the different datasets.

iTRAQ labelling, high pH reversed-phase separation and phosphopeptide enrichment of peptide samples. Desalted peptides were labelled with 4-plex iTRAQ reagents according to the manufacturer's instructions (AB Sciex, Foster City, CA). For each 1 mg peptide from each breast tumour sample, 10 units of labelling reagent were used. Peptides were dissolved in 300 μl of 0.5 M triethylammonium bicarbonate (TEAB) (pH 8.5) solution and labelling reagent was added in 700 μl of ethanol. After 1 h incubation, 1.5 ml of 0.05% TFA was added to stop the reaction. Differentially labelled peptides were mixed and subsequently desalted on 500 mg tC18 SepPak columns. The combined 4 mg iTRAQ samples per experiment were separated into 24 proteome fractions and 12 phosphoproteome fractions using a 4.6 mm × 250 mm column RP Zorbax 300 A ExtendC18 column (Agilent, 3.5 μm bead size) on an Agilent 1100 Series HPLC instrument by basic reversed-phase chromatography as described previously^46^. Peptides were separated according to their hydrophobicity using solvent A (2% acetonitrile, 5 mM ammonium formate, pH 10) and a nonlinear increasing concentration of solvent B (90% acetonitrile, 5 mM ammonium formate, pH 10). Phosphopeptides were enriched using Ni-NTA superflow agarose beads (Qiagen, #1018611) that were stripped of nickel with 100 mM EDTA and incubated in an aqueous solution of 10 mM FeCl_3_ (Sigma, 451649) as described previously^50^. For phosphopeptide enrichment a 80% acetonitrile/0.1% trifluoroacetic acid binding buffer and a 500 mM dibasic sodium phosphate, pH 7.0, (Sigma, S9763) elution buffer were used. Enriched samples were desalted on StageTips as described^50^ before analysis by LC–MS/MS.

Analysis of tumour samples by high performance liquid chromatography tandem mass spectrometry (LC–MS/MS). All peptides were separated with an online nanoflow Proxeon EASY-nLC 1000 UHPLC system (Thermo Fisher Scientific) and analyzed on a benchtop Orbitrap Q Exactive mass spectrometer (Thermo Fisher Scientific) equipped with a nanoflow ionization source (James A. Hill Instrument Services, Arlington, MA, USA). The LC system, column, and platinum wire to deliver electrospray source voltage were connected via a stainless-steel cross (360 μm, IDEX Health & Science, UH-906x). The column was heated to 50 °C using a column heater sleeve (Phoenix-ST) to prevent overpressurizing of columns during UHPLC separation. 10% of each global proteome sample in a 2 μl injection volume, or 50% of each phosphoproteome sample in a 4 ul injection volume was injected onto an in-house packed 20 cm × 75 um diameter C18 silica picofrit capillary column (1.9 μm ReproSil-Pur C18-AQ beads, Dr Maisch GmbH, r119.aq; Picofrit 10 um tip opening, New Objective, PF360-75-10-N-5). Mobile phase flow rate was 200 nl min^−1^, comprised of 3% acetonitrile/0.1% formic acid (Solvent A) and 90% acetonitrile /0.1% formic acid (Solvent B), and the 110-minute LC-MS/MS method consisted of a 10-min column-equilibration procedure, a 20-min sample-loading procedure, and the following gradient profile: (min:%B) 0:2; 1:6; 85:30; 94:60; 95;90; 100:90; 101:50; 110:50 (last two steps at 500 nl min^−1^ flowrate). Data-dependent acquisition was performed using Xcalibur QExactive v2.1 software in positive ion mode at a spray voltage of 2.00 kV. MS1 Spectra were measured with a resolution of 70,000, an AGC target of 3e6 and a mass range from 300 to 1,800 *m*/*z*. Up to 12 MS2 spectra per duty cycle were triggered at a resolution of 17,500, an AGC target of 5e4, an isolation window of 2.5 *m*/*z*, a maximum ion time of 120 msec, and a normalized collision energy of 28. Peptides that triggered MS2 scans were dynamically excluded from further MS2 scans for 20 s. Charge state screening was enabled to reject precursor charge states that were unassigned, 1, or >6. Peptide match was enabled for monoisotopic precursor mass assignment.

Protein-peptide identification, phosphosite localization, and quantitation. All MS data were interpreted using the Spectrum Mill software package v5.1 (for comparison with proteomes of human breast tumours from our previous study^14^) and v6.0 pre-release (Agilent Technologies, Santa Clara, CA, USA) co-developed by the authors. Similar MS/MS spectra acquired on the same precursor *m*/*z* within ± 45 s were merged. MS/MS spectra were excluded from searching if they failed the quality filter by not having a sequence tag length>0 (that is, minimum of two masses separated by the in-chain mass of an amino acid) or did not have a precursor MH+ in the range of 750–6,000. MS/MS spectra from were searched against a database consisting of RefSeq release 60 containing 31,767 human proteins, 24,821 mouse proteins, and an appended set of 85 common laboratory contaminant proteins (RefSeq.20130727-Human.20130730-MouseNR.mm13.contams). Scoring parameters were ESI-QEXACTIVE-HCD-v2, for whole proteome datasets, and ESI-QEXACTIVE-HCD-v3 parameters were for phosphoproteome datasets. All spectra were allowed ±20 p.p.m. mass tolerance for precursor and product ions, 40% minimum matched peak intensity, and trypsin allow P enzyme specificity with up to four missed cleavages. Fixed modifications were carbamidomethylation at cysteine. iTRAQ labelling was required at lysine, but peptide N-termini were allowed to be either labelled or unlabelled. Allowed variable modifications for whole proteome datasets were acetylation of protein N-termini, oxidized methionine, deamidation of asparagine, pyro-glutamic acid at peptide N-terminal glutamine, and pyro-carbamidomethylation at peptide N-terminal cysteine with a precursor MH+ shift range of −18 to 64 Da. Allowed variable modifications for phosphoproteome dataset were revised to disallow deamidation and allow phosphorylation of serine, threonine, and tyrosine with a precursor MH+ shift range of 0 to 272 Da.

Identities interpreted for individual spectra were automatically designated as confidently assigned using the Spectrum Mill autovalidation module to use target-decoy based FDR estimates to apply score threshold criteria via two-step strategies. For the whole proteome datasets thresholding was done at the spectral and protein levels. For the phosphoproteome datasets thresholding was done at the spectral level. In step 1, peptide autovalidation was done first and separately for each iTRAQ 4-plex experiment consisting of either 25 LC–MS/MS runs (whole proteome) or 13 LC–MS/MS runs (phosphoproteome) using an auto thresholds strategy with a minimum sequence length of 7(whole proteome) or 8 (phosphoproteome), automatic variable range precursor mass filtering, and score and delta Rank1–Rank2 score thresholds optimized to yield a spectral level FDR estimate for precursor charges 2 through 4 of <0.6% for each precursor charge state in each LC–MS/MS run. For precursor charges 5–6, thresholds were optimized to yield a spectral level FDR estimate of <0.3% across all runs per iTRAQ 4-plex experiment (instead of each run), to achieve reasonable statistics, since many fewer spectra are generated for the higher charge states.

In step 2 for the whole proteome datasets, protein polishing autovalidation was applied separately to each iTRAQ 4-plex experiment to further filter the PSM's using a target protein-level FDR threshold of zero. The primary goal of this step was to eliminate peptides identified with low scoring peptide spectrum matches (PSM's) that represent proteins identified by a single peptide, so-called ‘one-hit wonders'. After assembling protein groups from the autovalidated PSM's, protein polishing determined the maximum protein level score of a protein group that consisted entirely of distinct peptides estimated to be false-positive identifications (PSM's with negative delta forward-reverse scores). PSM's were removed from the set obtained in the initial peptide-level autovalidation step if they contributed to protein groups that have protein scores below the max false-positive protein score. In the filtered results each identified protein detected in an iTRAQ 4-plex experiment was comprised of multiple peptides unless a single excellent scoring peptide was the sole match. For the whole proteome datasets the above criteria yielded FDR of <0.5% at the peptide-spectrum match level and <0.8% at the distinct peptide level for each iTRAQ 4-plex experiment. After assembling proteins with all the PSMs from all the iTRAQ 4-plex experiments together the aggregate FDR estimates were 0.42% at the at the peptide-spectrum match level, 1.5% at the distinct peptide level, and <0.01% (1/11,372) at the protein group level. Since the protein level FDR estimate neither explicitly required a minimum number of distinct peptides per protein nor adjusted for the number of possible tryptic peptides per protein, it may underestimate false positive protein identifications for large proteins observed only on the basis of multiple low scoring PSMs.

In calculating scores at the protein level and reporting the identified proteins, redundancy was addressed in the following manner: the protein score was the sum of the scores of distinct peptides. A distinct peptide was the single highest scoring instance of a peptide detected through an MS/MS spectrum. MS/MS spectra for a particular peptide may have been recorded multiple times, (that is, as different precursor charge states, in adjacent bRP fractions, modified by deamidation at Asn or oxidation of Met, or different phosphosite localization) but were still counted as a single distinct peptide. When a peptide sequence >8 residues long was contained in multiple protein entries in the sequence database, the proteins were grouped together and the highest scoring one and its accession number were reported. In some cases when the protein sequences were grouped in this manner there were distinct peptides that uniquely represented a lower scoring member of the group (isoforms, family members, and different species). Each of these instances spawned a subgroup. Multiple subgroups were reported and counted towards the total number of proteins, and were given related protein subgroup numbers (for example, 3.1 and 3.2: group 3, subgroups 1 and 2). To better dissect the tumour/stroma (human/mouse) origin of orthologous proteins in this xenograft experiment, the inclusion of peptides contributing to each subgroup was restricted by enabling the subgroup-specific (SGS) option in Spectrum Mill. Only subgroup-specific peptide sequences were counted toward each subgroup's count of distinct peptides and protein level TMT quantitation. The SGS option omits peptides that are shared between subgroups. If evidence for BOTH human and mouse peptides from an orthologous protein were observed, then peptides that cannot distinguish the two (shared) were ignored. However, the peptides shared between species were retained if there was specific evidence for only one of the species, thus yielding a single subgroup attributed to only the single species consistent with the specific peptides. Furthermore, if all peptides observed for a protein group were shared between species, thus yielding a single subgroup composed of indistinguishable species, then all peptides were retained (the column in Supplementary Data 3 numSpeciesPresentR1 will have a value of 2 in such cases). Assembly of confidently identified PSM's yielded 20,480 total protein subgroups from 11,372 protein groups. Human and mouse ortholog proteins were typically arranged into individual subgroups.

In step 2 for the phosphoproteome datasets a phosphosite table were assembled with columns for individual iTRAQ 4-plex experiments and rows for individual phosphosites. PSM's were combined into a single row for all non-conflicting observations of a particular phosphosite. (that is, different missed cleavage forms, different precursor charges, confident and ambiguous localizations, different sample handling modifications). For related peptides neither observations with a different number of phosphosites nor different confident localizations were allowed to be combined. Selecting the representative peptide from the combined observations was done such that once confident phosphosite localization was established, higher identification scores and longer peptide lengths are preferable. After assembling the phosphosite table a polishing step was applied to further filter the phosphosites with the primary goal of eliminating phosphosites with representative peptides identified through low scoring peptide spectrum matches (PSM's) that were observed in only a few experiments. The initial table of representative peptides for 82,030 phosphosites had an aggregate FDR of 3.3% at phosphosite-level. The table was sorted by identification score and then by number of iTRAQ 4-plex experiments in which the phosphosite was observed. The cumulative FDR trend showed inflection points at an identification score of ∼8. Phosphosites with an identification score<8.0 observed in <3/9 experiments were therefore removed, yielding 68,385 phosphosites with an aggregate FDR of 0.34% at the phosphosite level. While the Spectrum Mill identification score is based on the number of matching peaks, their ion type assignment, and the relative height of unmatched peaks, the phosphosite localization score is the difference in identification score between the top two localizations. The score threshold for confident localization (>1.1) essentially corresponds to at least 1 b or y ion located between two candidate sites that has a peak height 10% of the tallest fragment ion (neutral losses of phosphate from the precursor and related ions as well as immonium and iTRAQ reporter ions are excluded from the relative height calculation). The ion type scores for b-H3PO4, y-H3PO4, b-H2O, and y-H2O ion types are all set to 0.5. This prevents inappropriate confident localization assignment when a spectrum lacks primary b or y ions between two possible sites but contains ions that can be assigned as either phosphate loss ions for one localization or water loss ions for another localization. In aggregate, 66.3% of the reported phosphosites were fully localized to a particular serine, threonine, or tyrosine residue.

Relative abundances of proteins and phosphosites were determined in Spectrum Mill using iTRAQ reporter ion intensity ratios from each PSM. A protein-level or phosphosite-level iTRAQ ratio was calculated as the median of all PSM level ratios contributing to a protein subgroup or phosphosite remaining after excluding those PSM's lacking an iTRAQ label, having a negative delta forward-reverse score (half of all false-positive identifications), or having a precursor ion purity<50% (MS/MS has significant precursor isolation contamination from co-eluting peptides). Unless stated otherwise for a particular analysis, the following considerations apply to the tumour/stroma (human/mouse) origin of a protein in this xenograft experiment. For the proteome dataset, only PSM's from subgroup-specific peptide sequences contributed to the protein level quantitation (see protein subgrouping description above). A protein detected with all contributing PSM's shared between human and mouse was considered to be human. For the phosphoproteome dataset, a phosphosite was considered to be mouse if the contributing PSM's were distinctly mouse and human if they were either distinctly human or shared between human and mouse. A 2-component Gaussian mixture model-based normalization approach was used to centre the distribution of iTRAQ log-ratios around zero to nullify the effect of differential protein loading and/or systematic MS variation^14^. Downstream analyses presented in the main figures were restricted to proteins/phosphosites quantified in at least 10 out of the 24 samples with non-missing values (Supplementary Fig. 3), with the exception of the previously described mRNA-protein correlation analysis^13^ requiring quantification in 30%, or 8 out of 24, PDX samples. Specific filtering procedures are noted in descriptions of the relevant methods.

### Bioinformatics analyses.

#### Cross data-type integration

All gene names were converted to HUGO Gene Nomenclature Committee's approved gene names for comparison across levels and datasets. For mRNA and protein expressions, expression values were collapsed across transcripts or isoforms to the corresponding gene using the highest mean when there were two transcripts or isoforms, or the value with the highest connectivity when there were more than three transcripts or isoforms as implemented in the WGCNA R package^51^.

#### mRNA-protein correlation

Spearman correlations between normalized RSEM values and protein quantifications were calculated for genes that were observed in at least 30% of samples for both RNA-seq and mass-spectrometry as previously described. KEGG pathway enrichment analysis of the correlation was carried out using a Kolmogorov–Smirnov test, and *P* values were adjusted using the Benjamini-Hochberg procedure.

#### Proteomic clustering and subtyping

We first applied filtering for protein and phosphosite markers observed in at least 10 samples and sufficient deviations across samples. We used a 2 s.d. threshold for iTRAQ proteome and 2.5 s.d. for the phosphoproteome. We applied the same protein marker to conduct LFQ proteome and PDX vs. human proteome co-clustering. For the co-clustering with human proteome, we further selected for markers that had higher than 2 s.d.'s in the merged proteome and showed non-differential expression between human and PDX (FDR>0.3, *t*-test). The subsequent hierarchical clustering was conducted using the complete agglomeration method of hclust as implemented in the heatmap2 R package.

#### Differential expression analysis

Differential expression testing of each protein in the LFQ and iTRAQ datasets was conducted using the student's *t*-test, and *P* values were adjusted using the Benjamini-Hochberg procedure. For gene set enrichment analysis, we conducted the Wilcoxon Rank Sum Test to test for changes in the t-statistics ranks of protein members in each of the KEGG signalling pathway, and again adjusted *P* values using the Benjamini-Hochberg procedure.

#### Druggable genes and mutations

We compiled a list of druggable genes based on extensive curation of public databases: Tumor Alterations Relevant for GEnomics-driven Therapy (TARGET, version 3, assessed on 6/15/2015), Personalized Cancer Therapy (PCT, assessed on 3/15/2015), GDKD (Gene-Drug Knowledge Database, version 11.0, assessed on 4/10/2015), CancerDR (assessed on 2/6/2015), My Cancer Genome (assessed on 9/11/2014), and DrugBank (assessed on 9/21/2015). We curated the list based on evidence level and literature, as well as IC50 data when available. The final list used for the analysis included 76 druggable genes (Supplementary Data 15).

#### Druggable outlier analysis

To discover expression outliers, we utilized a strategy incorporating multiple steps: first, we limited our search to genes that are in the druggable gene list. We then narrowed down the list to genes that are observed in at least 10 samples in the dataset under investigation. Outlier expressions were defined as values that are greater than 1.5 interquartile ranges (IQRs) above the third quartile (Q3). To rank order outlier expression for each gene, we calculated an outlier score defined as:


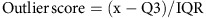


By definition, genes with outlier score greater than 1.5 are considered as expression outliers. Outlier score for each gene were ranked within the sample to select the most promising druggable targets. For CNV outliers, we required them to have outlier scores above 1 in at least another expression level. For validation of the proteomic druggable outliers, we counted the numbers of the same protein or phosphosite outliers observed in the parallel-processed cohort of 77 human breast cancer samples. Due to the lower numbers in this larger cohort, we considered both proteins with outlier score greater than 1 and the top 2 outliers of each human sample as validating outliers.

#### Pathway activation analysis

We first collapsed the phosphosites to gene-level phosphorylation values by averaging the phosphosite expressions observed for each gene. Then, we converted the phosphoproteomic expression values from iTRAQ to modified *z*-scores normalized against the cohort as described in Hoaglin *et al*. (How to Detect and Handle Outliers). We then used the Wilcoxon Rank Sum Test to test for changes in the phosphorylation *z*-score ranks of protein members in each of the KEGG signalling pathway. The resulting *P* values were adjusted using the Benjamini–Hochberg procedure.

### Data availability

All mass spectra, in the original instrument vendor format, contributing to this study may be downloaded from: https://cptac-data-portal.georgetown.edu/cptacPublic/ for the study name: TCGA Breast Cancer.

## Additional information

**How to cite this article:** Huang, K.-l. *et al*. Proteogenomic integration reveals therapeutic targets in breast cancer xenografts. *Nat. Commun.* **8**, 14864 doi: 10.1038/ncomms14864 (2017).

**Publisher's note:** Springer Nature remains neutral with regard to jurisdictional claims in published maps and institutional affiliations.

## Supplementary Material

Supplementary Information Supplementary Figures

Supplementary Data 1 Clinical information of the 24 originating breast tumors included in this study.

Supplementary Data 2 The coverage of global profiling of the 24 breast cancer patient-derived xenografts.

Supplementary Data 3 iTRAQ global proteome analysis of human proteins.

Supplementary Data 4 LFQ global proteome analysis of human proteins.

Supplementary Data 5 iTRAQ global phosphosite analysis.

Supplementary Data 6 PAM50 gene markers used for the hierarchical clustering analysis in the resulting order.

Supplementary Data 7 iTRAQ protein markers used for the hierarchical clustering analysis in the resulting order.

Supplementary Data 8 LFQ protein markers used for the hierarchical clustering analysis in the resulting order.

Supplementary Data 9 iTRAQ mouse stromal protein markers used for the hierarchical clustering analysis in the resulting order.

Supplementary Data 10 iTRAQ phosphosite markers used for the hierarchical clustering analysis in the resulting order.

Supplementary Data 11 Differential expression analysis between 24 PDX and 77 breast tumors. The top 133 human protein markers with t-test FDR >= 0.3 were selected for joint proteomic clustering analysis.

Supplementary Data 12 Differential expression analysis of basal and luminal B breast tumor PDXs.

Supplementary Data 13 Significantly differentially-expressed Reactome pathways (FDR >= 0.01) identified through differential expression of basal and luminal B breast tumor PDXs using Reactome pathways.

Supplementary Data 14 Significantly activated pathways (FDR >= 0.01) identified through pathway phosphorylation enrichment analysis.

Supplementary Data 15 Potentially druggable genes and their corresponding drugs.

Supplementary Data 16 Identified druggable events in 24 breast cancer patient-derived xenografts across DNA, RNA, and protein levels.
