# Corrigendum: Proteogenomic integration reveals therapeutic targets in breast cancer xenografts

**DOI:** 10.1038/ncomms15479

**Published:** 2017-04-25

**Authors:** Kuan-lin Huang, Shunqiang Li, Philipp Mertins, Song Cao, Harsha P. Gunawardena, Kelly V. Ruggles, D. R. Mani, Karl R. Clauser, Maki Tanioka, Jerry Usary, Shyam M. Kavuri, Ling Xie, Christopher Yoon, Jana W. Qiao, John Wrobel, Matthew A. Wyczalkowski, Petra Erdmann-Gilmore, Jacqueline E. Snider, Jeremy Hoog, Purba Singh, Beifang Niu, Zhanfang Guo, Sam Qiancheng Sun, Souzan Sanati, Emily Kawaler, Xuya Wang, Adam Scott, Kai Ye, Michael D. McLellan, Michael C. Wendl, Anna Malovannaya, Jason M. Held, Michael A. Gillette, David Fenyö, Christopher R. Kinsinger, Mehdi Mesri, Henry Rodriguez, Sherri R. Davies, Charles M. Perou, Cynthia Ma, R. Reid Townsend, Xian Chen, Steven A. Carr, Matthew J. Ellis, Li Ding

Nature Communications 8: Article number: 14864; DOI: 10.1038/ncomms14864 (2017)); Published: 03 28 2017; Updated: 04 25 2017

The original version of this Article contained a typographical error in the spelling of the author Beifang Niu, which was incorrectly given as Beifung Niu. This has now been corrected in both the PDF and HTML versions of the Article.

