# Supplementary material for: Proteogenomic integration reveals therapeutic targets in breast cancer xenografts
Source: Nat Commun. 2017 Mar 28;8:14864. doi: 10.1038/ncomms14864 (PMC5379071; doi:10.1038/ncomms14864)
Supplement: Supplementary Information — Supplementary Figures [file ncomms14864-s1.pdf]

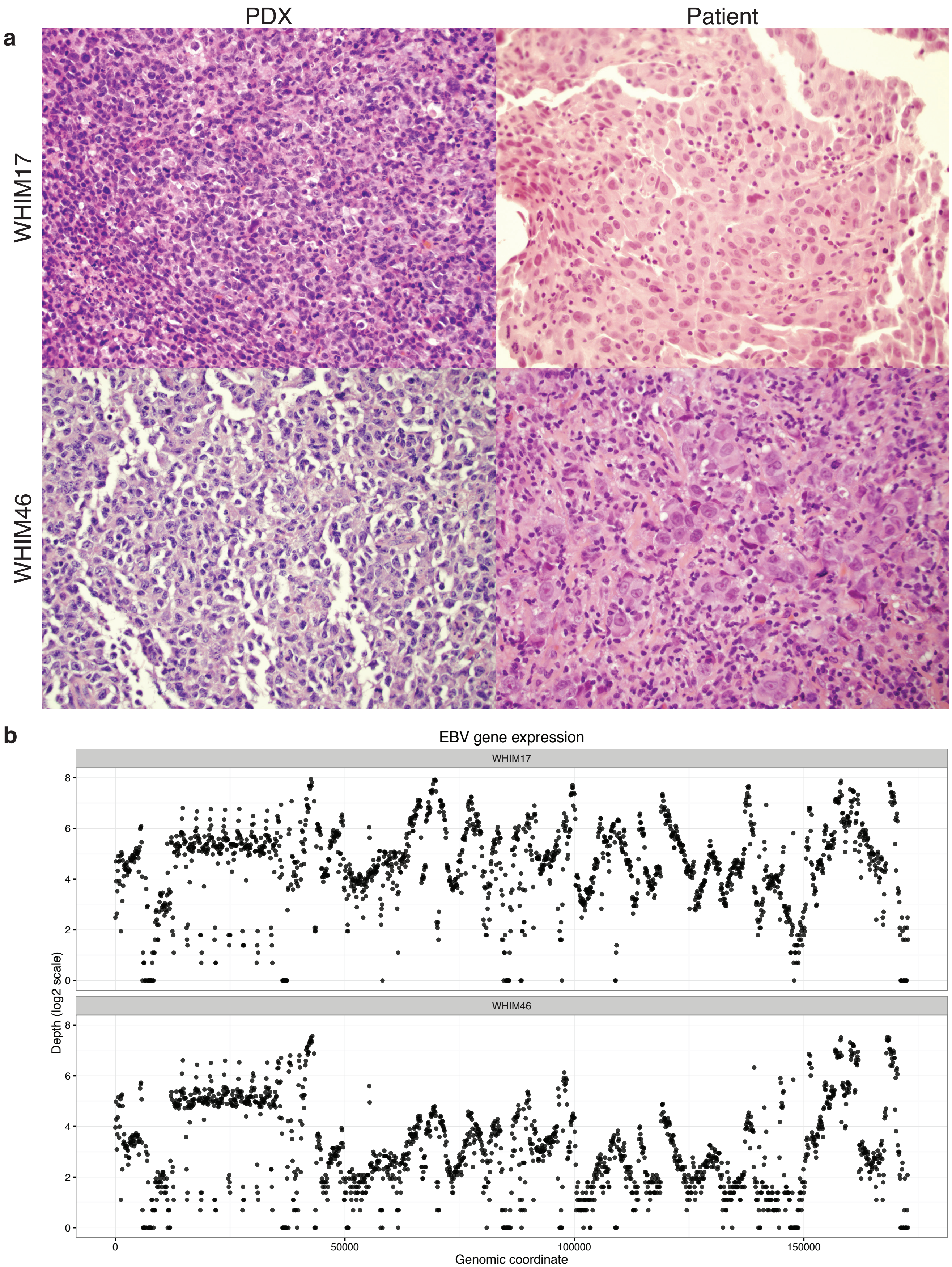

Supplementary Figure 1. Histology and EBV status of WHIM17 and WHIM46. (a) H&E stained histology slides of WHIM17, WHIM46, and their corresponding tumors in patient samples. (b) Gene expression levels across the EBV genome in WHIM17 and WHIM46.

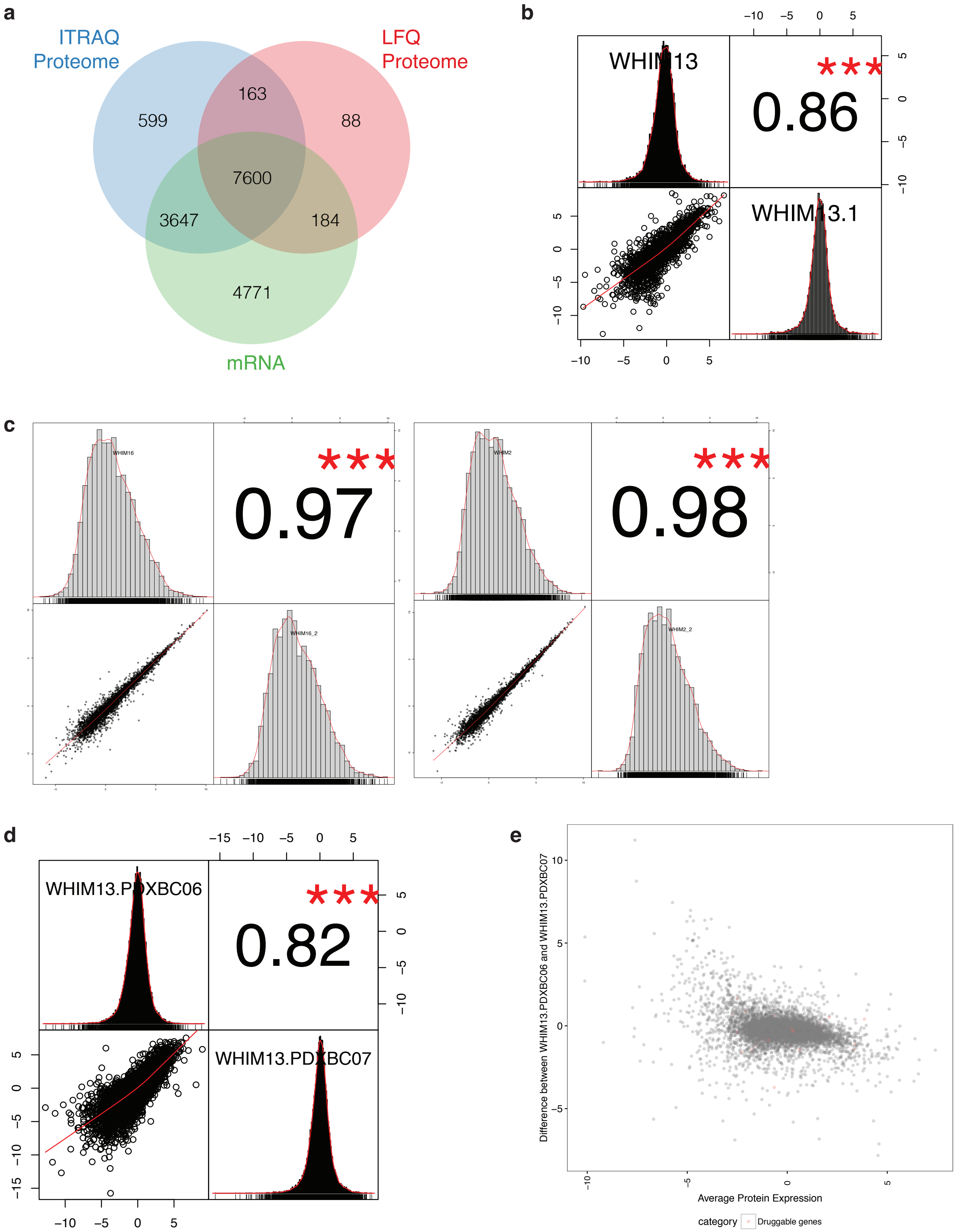

Supplementary Figure 2. Comprehensive coverage and technical reproducibility of proteomic data. (a) Overlap of detected genes with quantitative information in the mRNA, LFQ proteome, and iTRAQ proteome datasets. 7,600 genes were successfully quantified in all three datasets. (b) Protein expression correlations of technical replicates in the iTRAQ proteome dataset (WHIM13). (c) Protein expression correlations of technical replicates in the LFQ proteome dataset (WHIM2 and WHIM16). (d) Phosphosite expression correlations of technical replicates in the iTRAQ phosphoproteome dataset. (e) The bland-altman plot showing the difference between two technical replicates and average expression of protein markers in the iTRAQ proteome dataset (WHIM13). The red points indicate druggable proteins used in the druggable outlier analysis. ***: P value < 0.001.

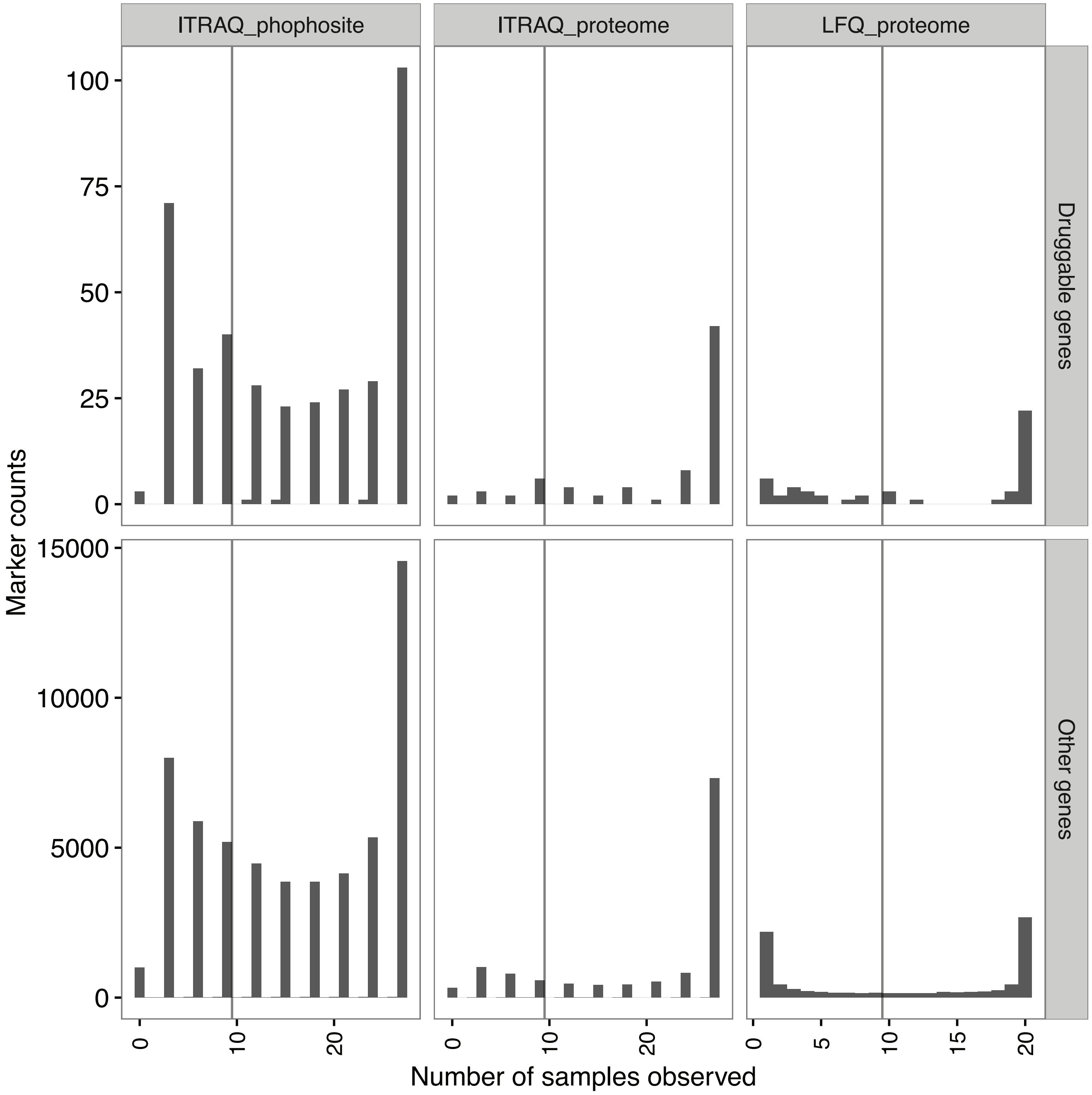

Supplementary Figure 3. Count distribution of number of samples where the protein or phosphosite marker is quantified in the iTRAQ phosphosite dataset, iTRAQ proteome dataset, and LFQ proteome dataset, separated by markers of druggable genes and all other genes. The markers to the right of the grey vertical lines are observed in more than 10 samples in the corresponding dataset.

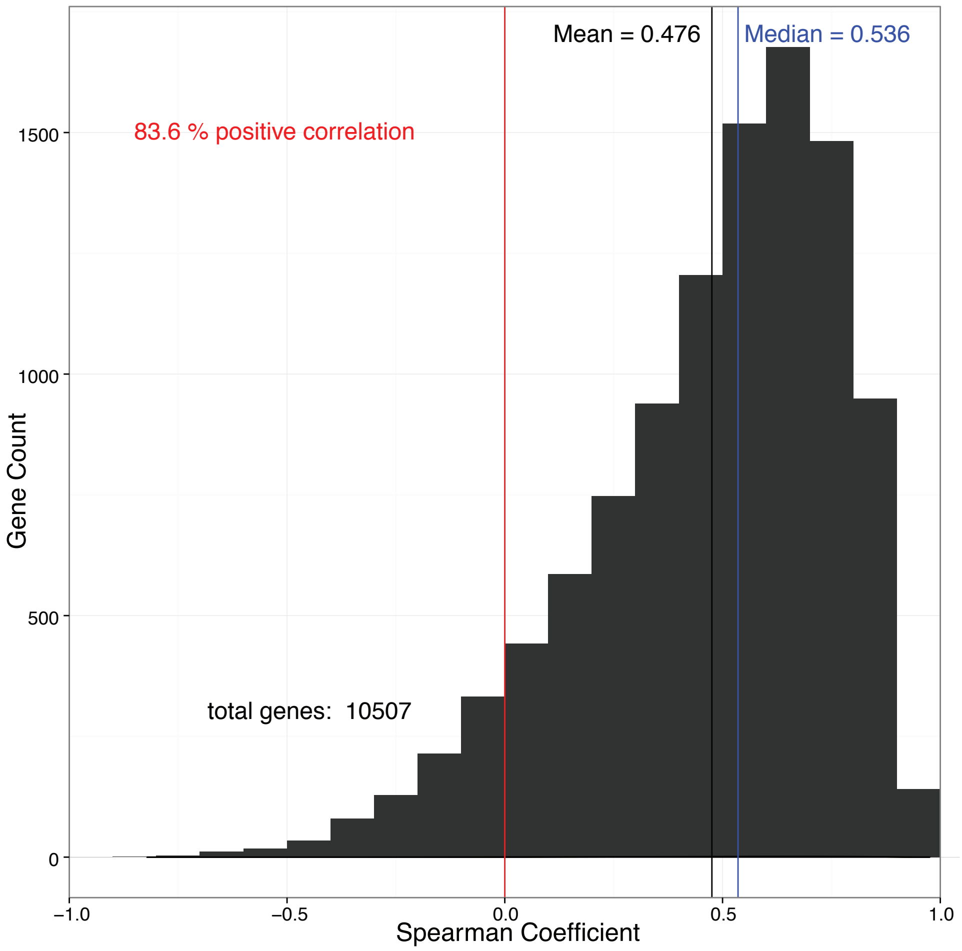

Supplementary Figure 4. Distribution of mRNA-protein expression correlation using RSEM and iTRAQ protein expression.

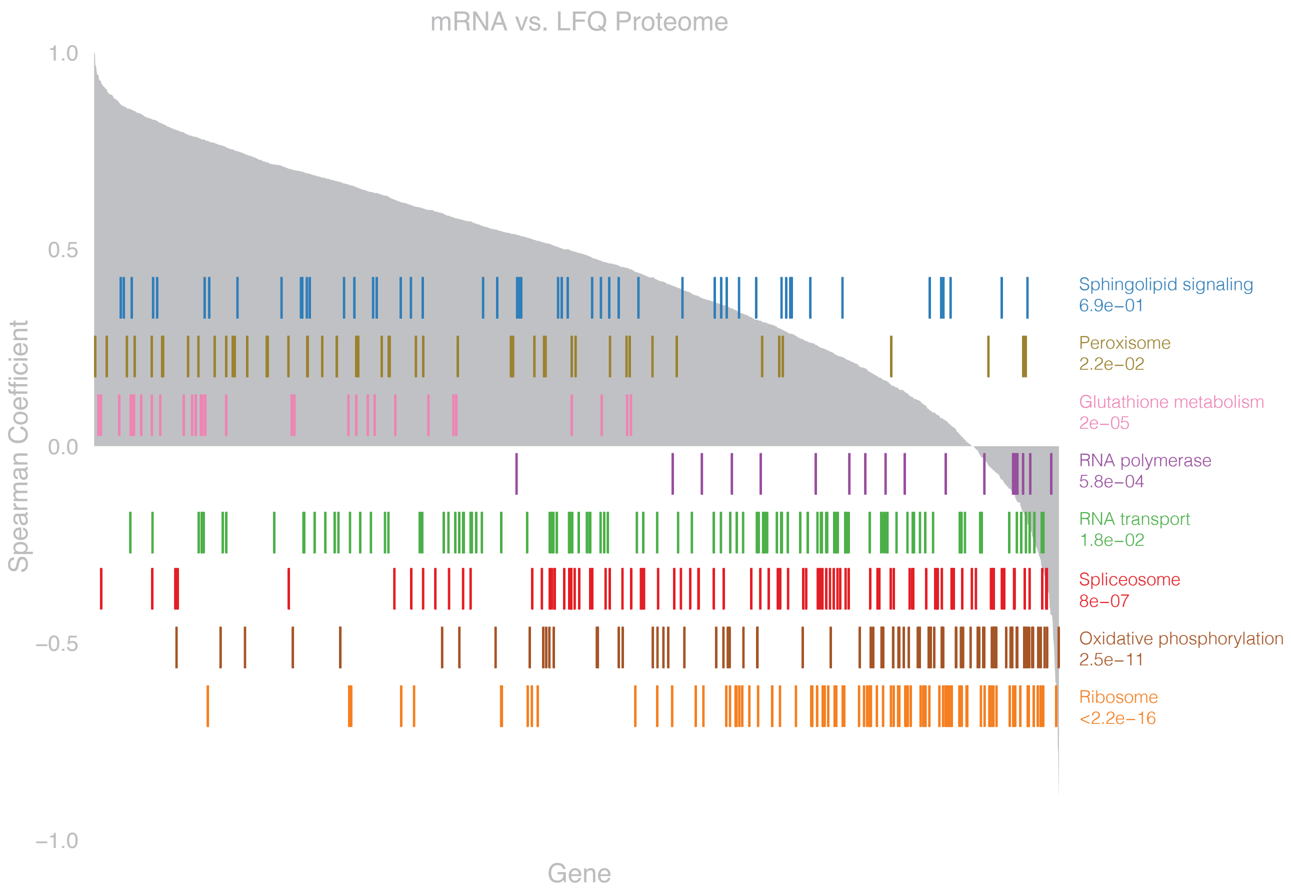

Supplementary Figure 5. Correlation between mRNA and LFQ protein expression levels and pathway enrichment analysis.

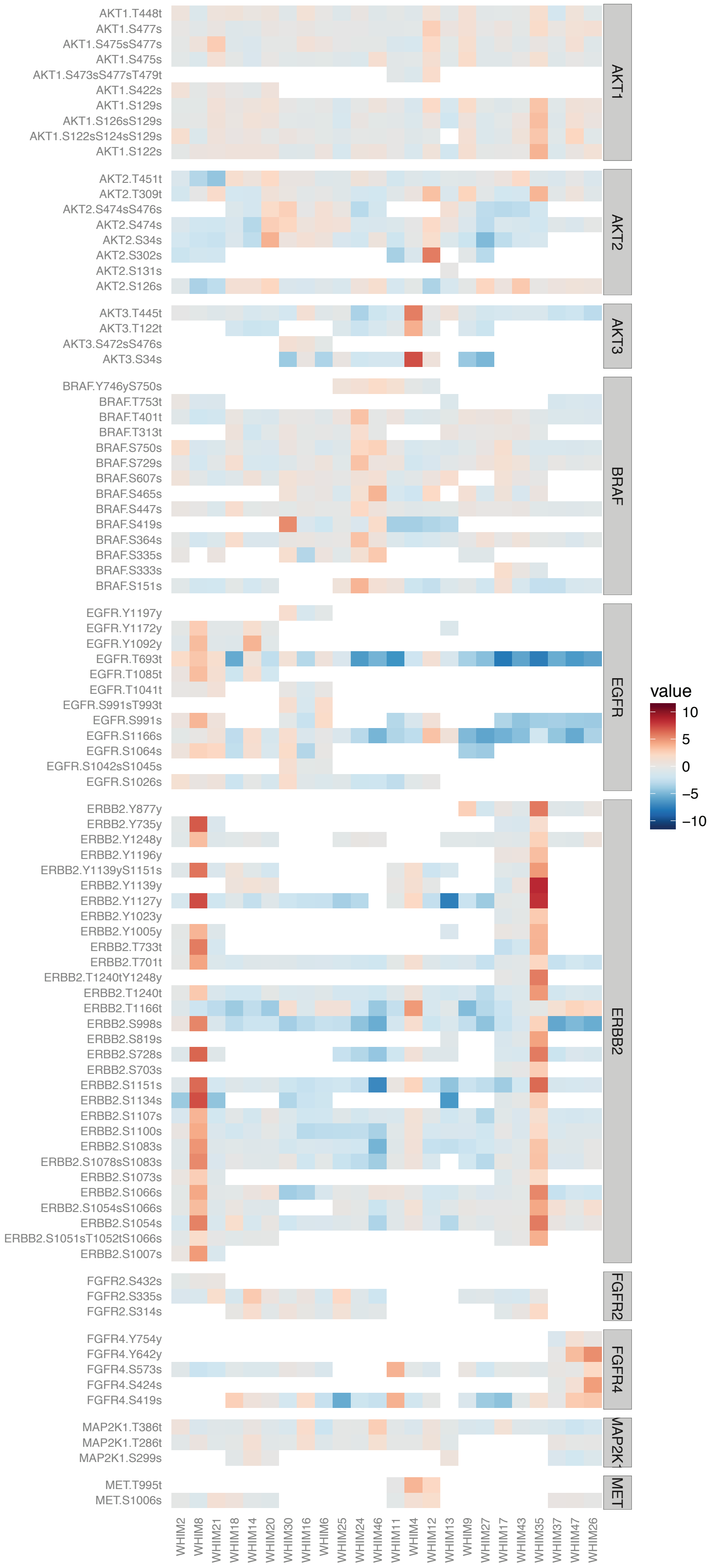

Supplementary Figure 6. Phosphosite expression levels of breast cancer-related genes.

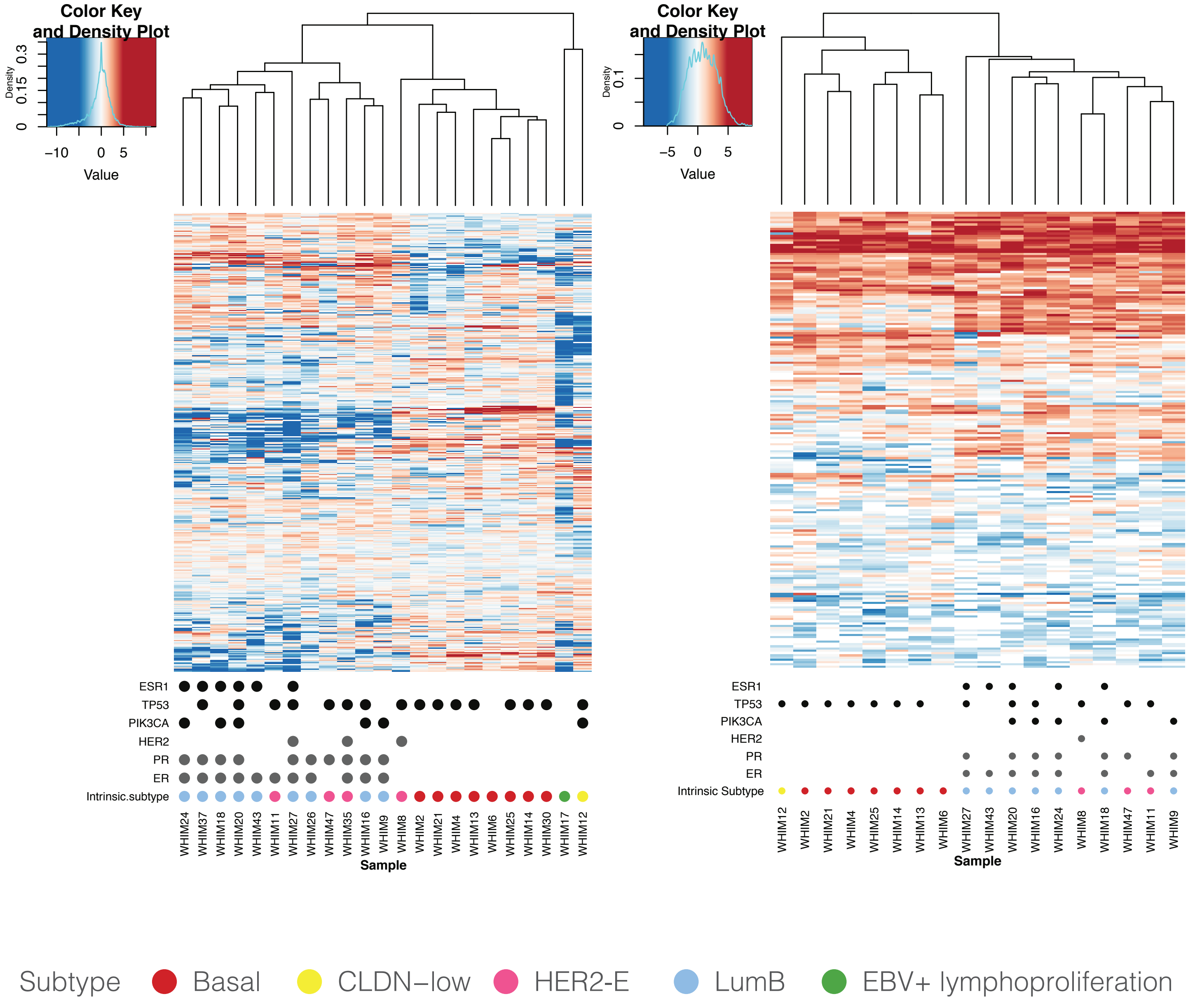

Supplementary Figure 7. Unsupervised clustering of proteomes using the markers identified through iTRAQ proteomic analysis. (a) Transcriptome clustering. (b) Proteomic clustering based on the LFQ proteome.

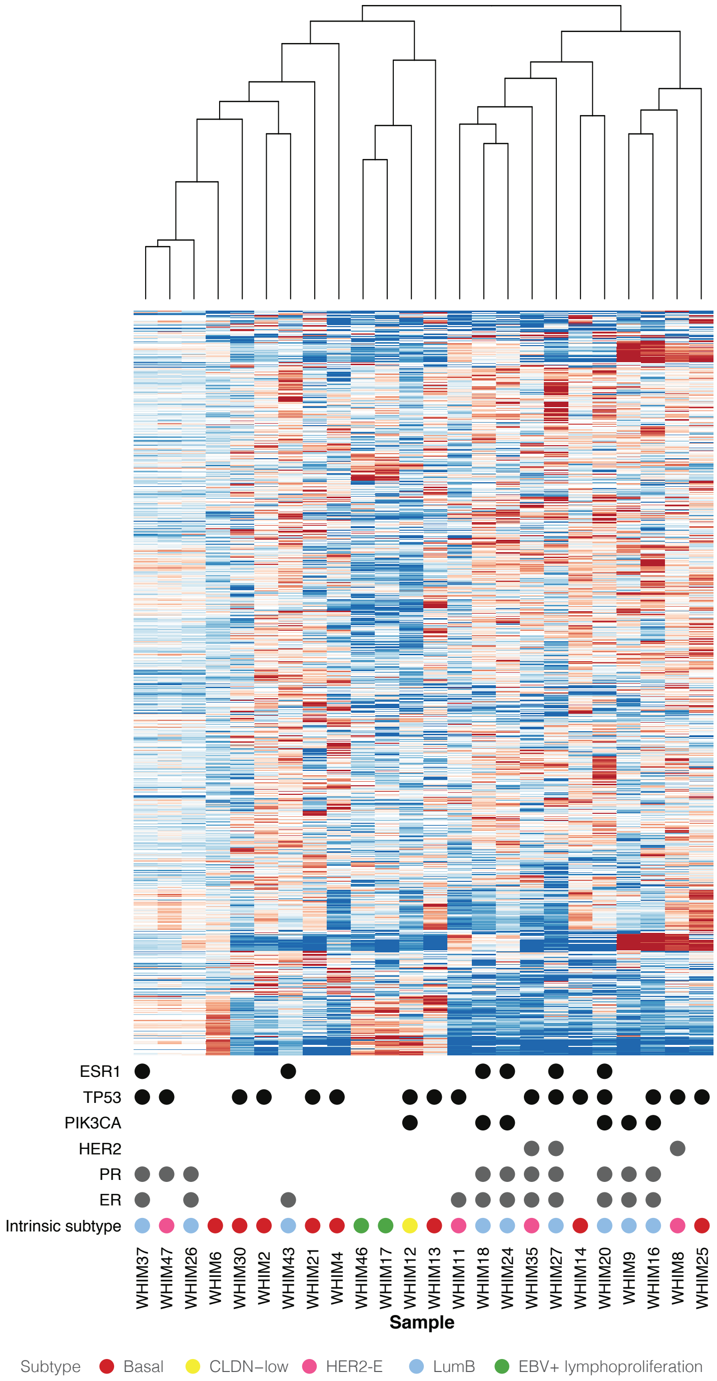

Supplementary Figure 8. Unsupervised clustering of the mouse stromal proteome identified in the iTRAQ dataset.

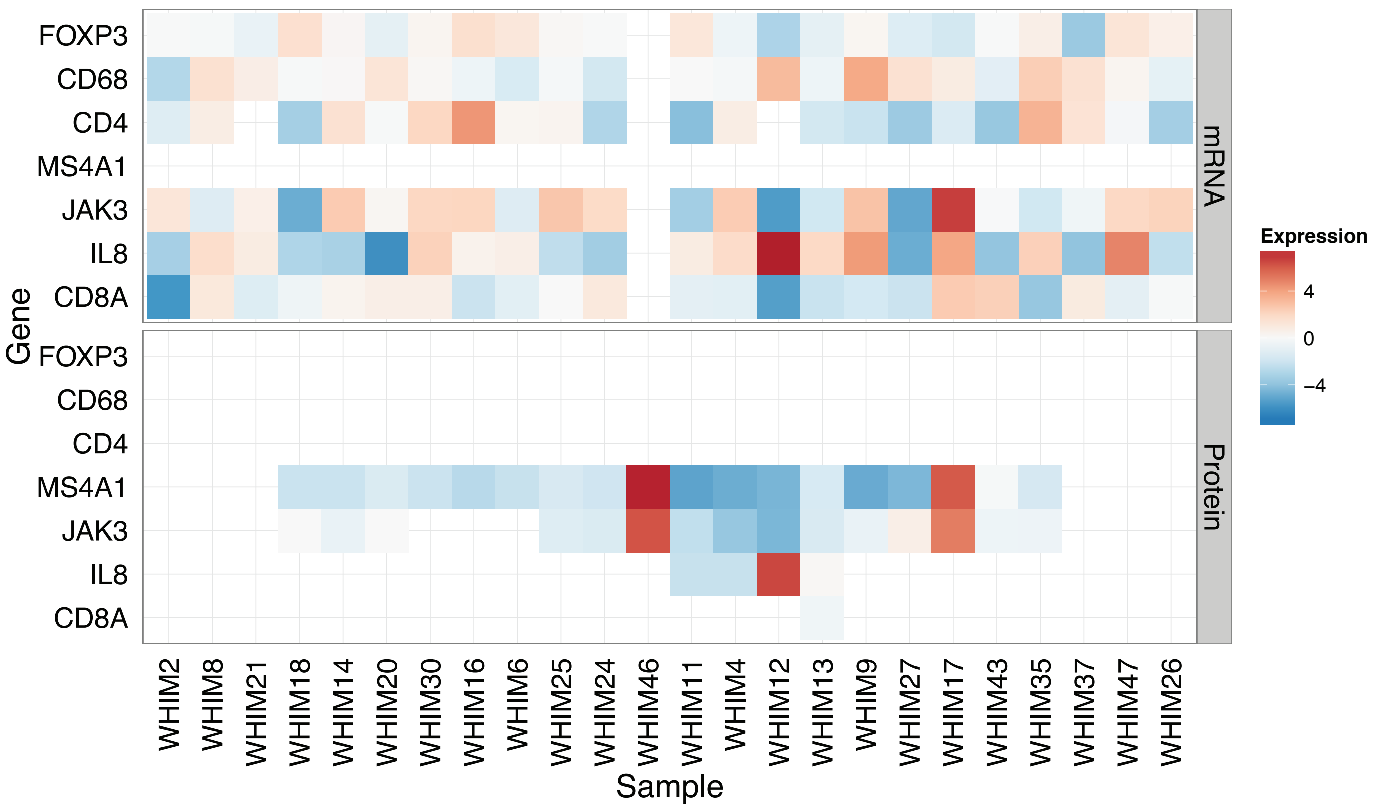

Supplementary Figure 9. Gene expression at the mRNA and iTRAQ protein levels of lymphoid lineage markers across PDX models, including FOXP3, CD68, CD4, MS4A1, JAK3, IL8, CD8A.

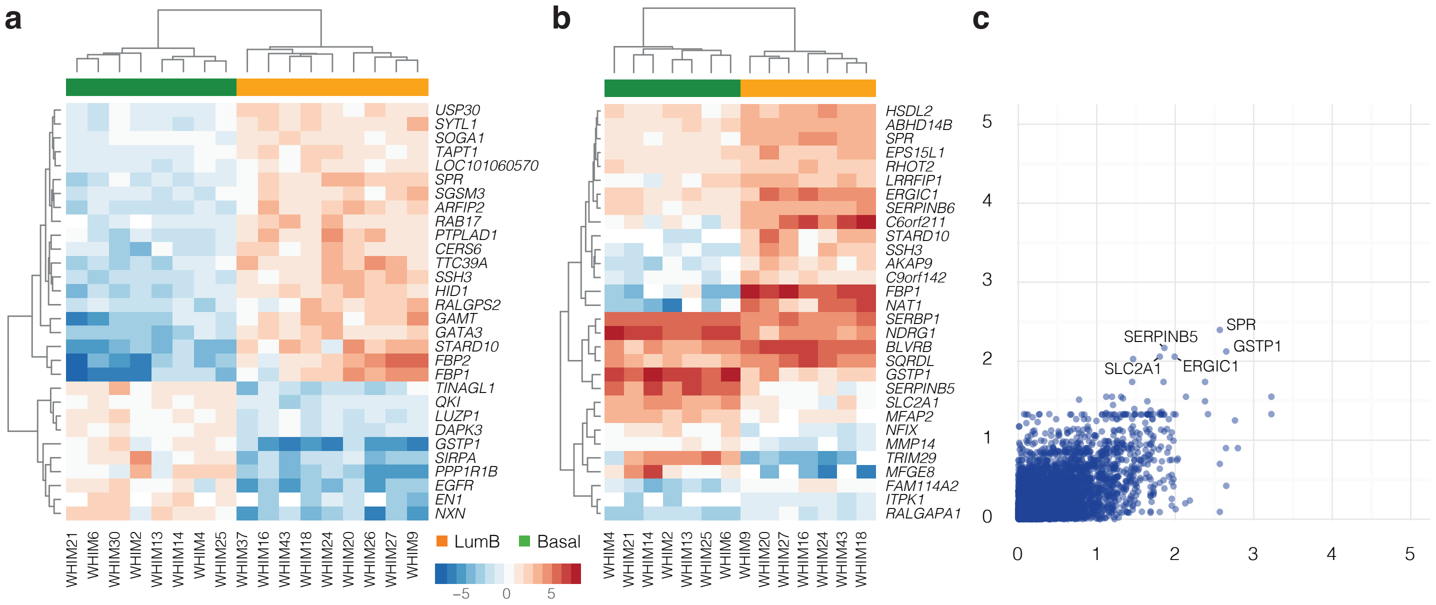

Supplementary Figure 10. Differentially expressed proteins in basal and luminal B breast cancer xenografts. (a) The top 30 most differentially expressed proteins in the ITRAQ proteome dataset. (b) The top 30 most differentially expressed proteins in the LFQ proteome dataset. (c) qqplot showing the –logFDR values of the differentially-expressed proteins in both datasets.

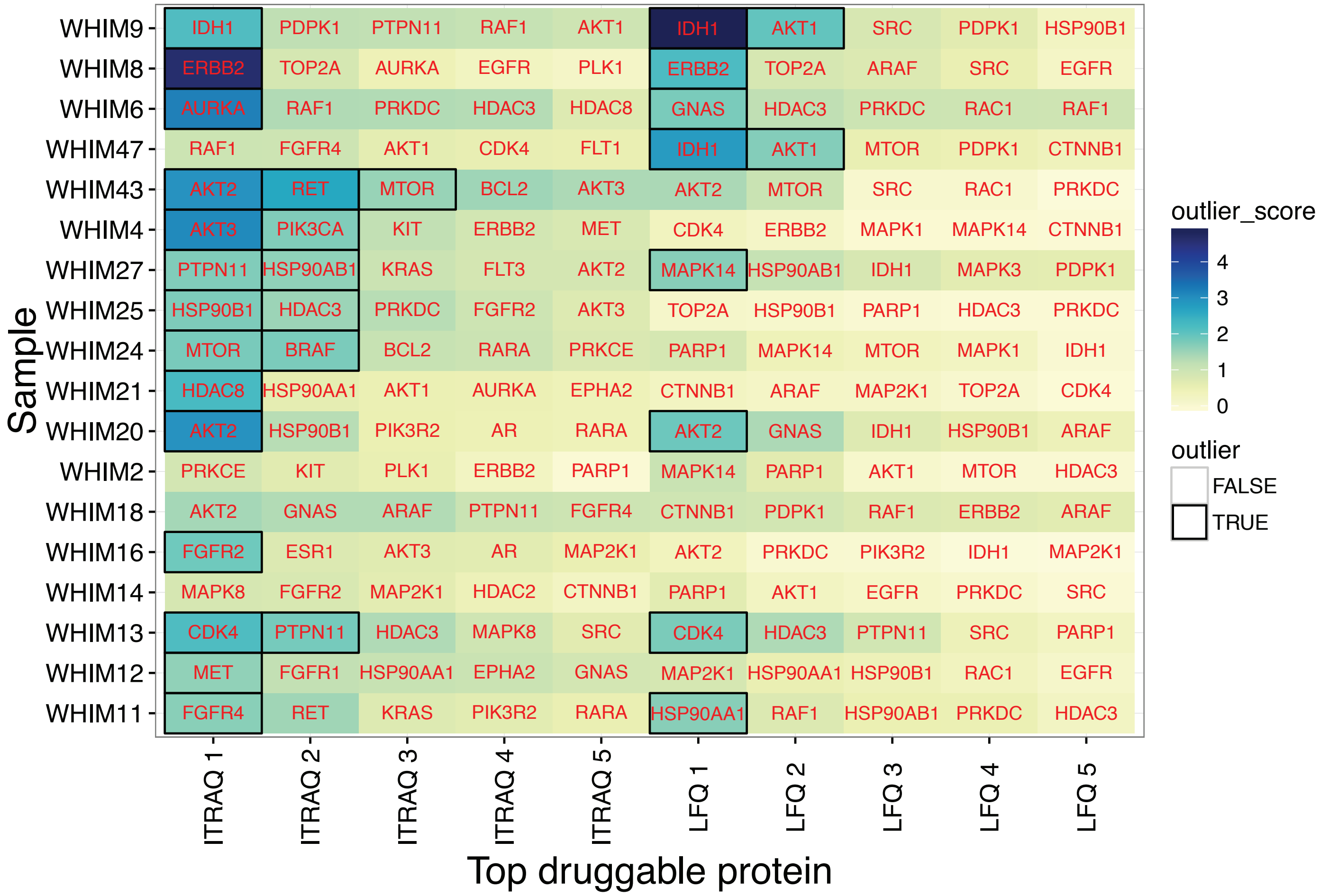

Supplemtary Figure 11. Top druggable protein expression outliers in the ITRAQ and LFQ proteome.

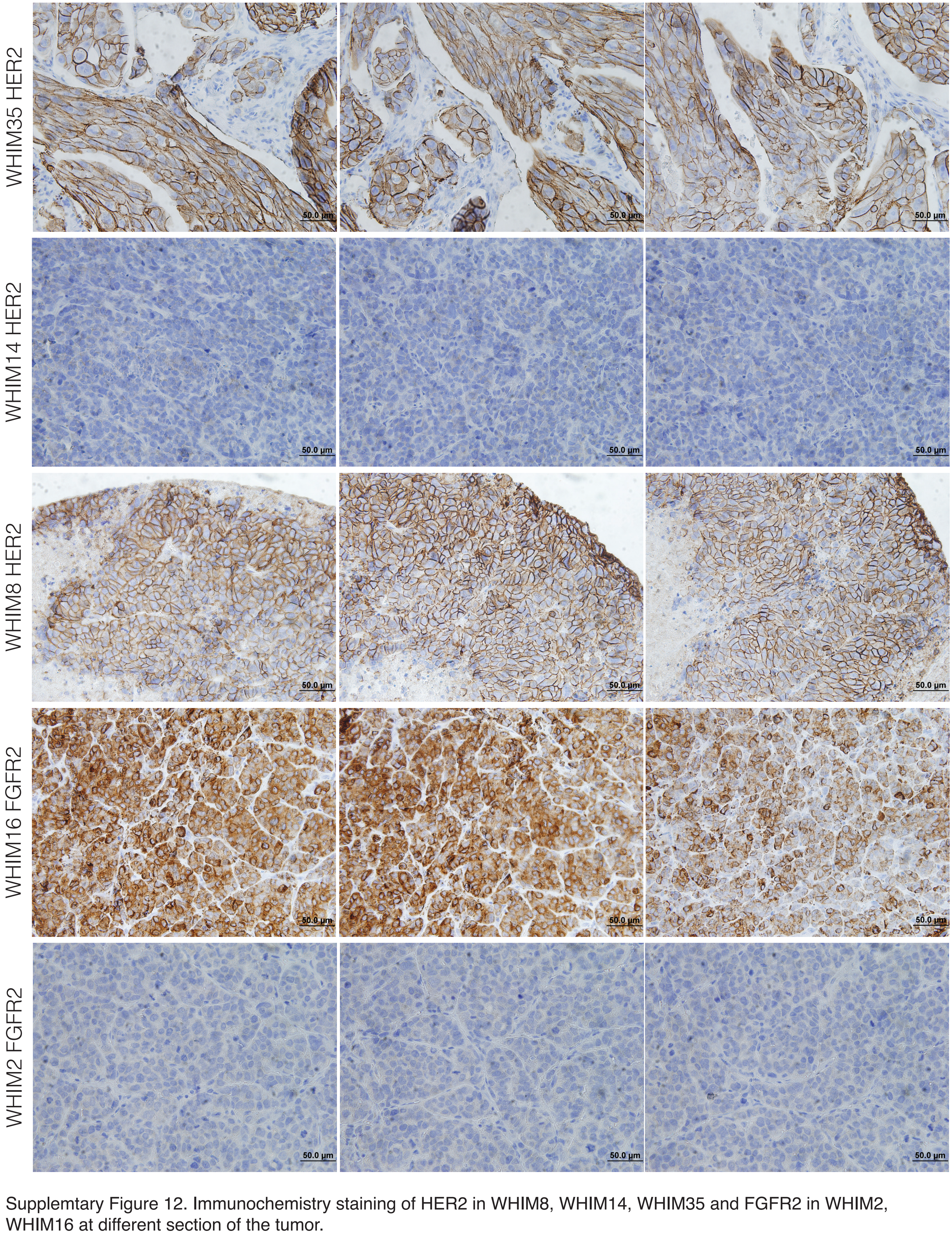

Supplemtary Figure 12. Immunochemistry staining of HER2 in WHIM8, WHIM14, WHIM35 and FGFR2 in WHIM2, WHIM16 at different section of the tumor.

Supplemental Figure 13

Annotated MS/MS spectra of the representative peptide for each of the outlier phosphorylation sites of the candidate druggable genes. See methods section for detailed description of the selection of the representative peptide from the multiple observations of a particular phosphosite across all 9 iTRAQ experiments. The phosphorylated amino acid is lowercase.

AKT1	S475s	RPHFPQFSYsASGTA
AKT1	S475s S477s	RPHFPQFSYsAsGTA
AKT3	S120s	MNCsPTSQIDNIGEEEMDASTTHHK

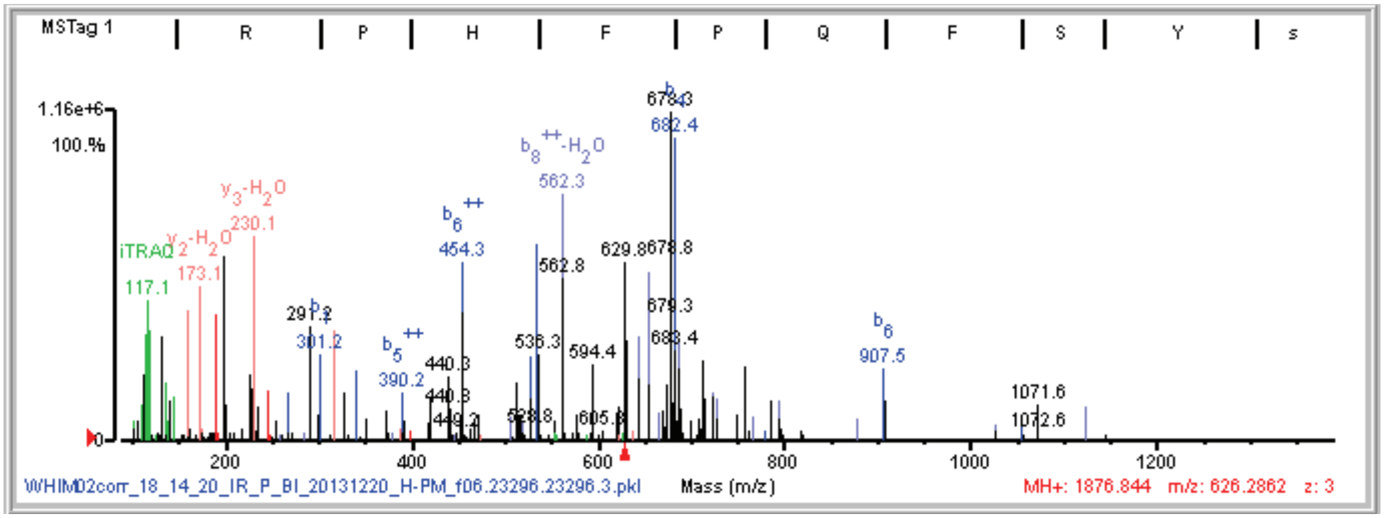

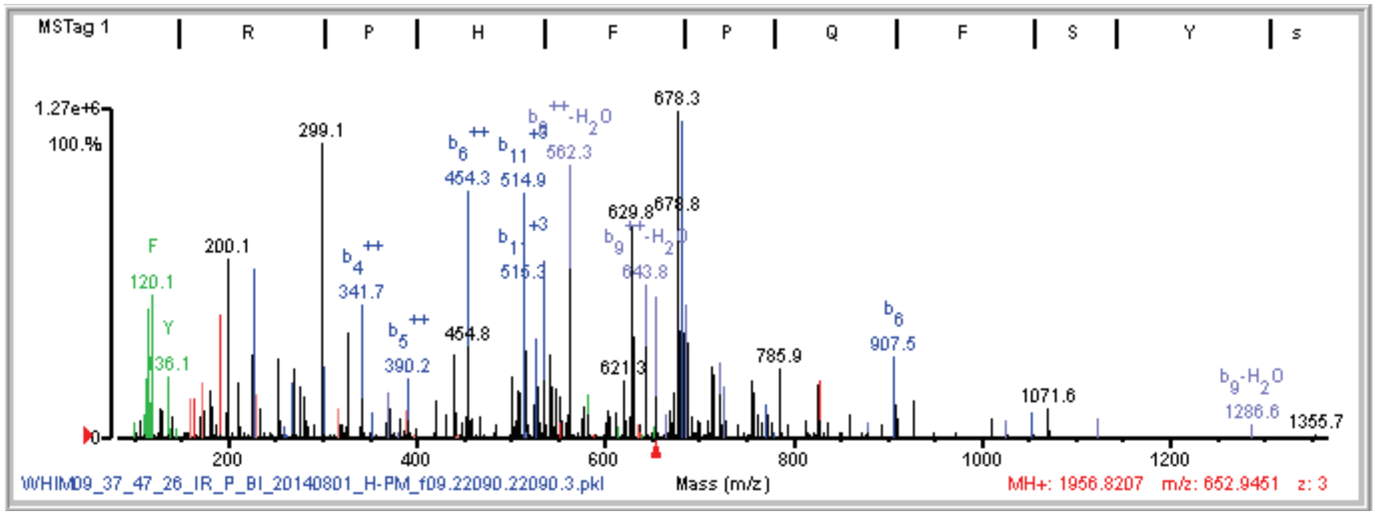

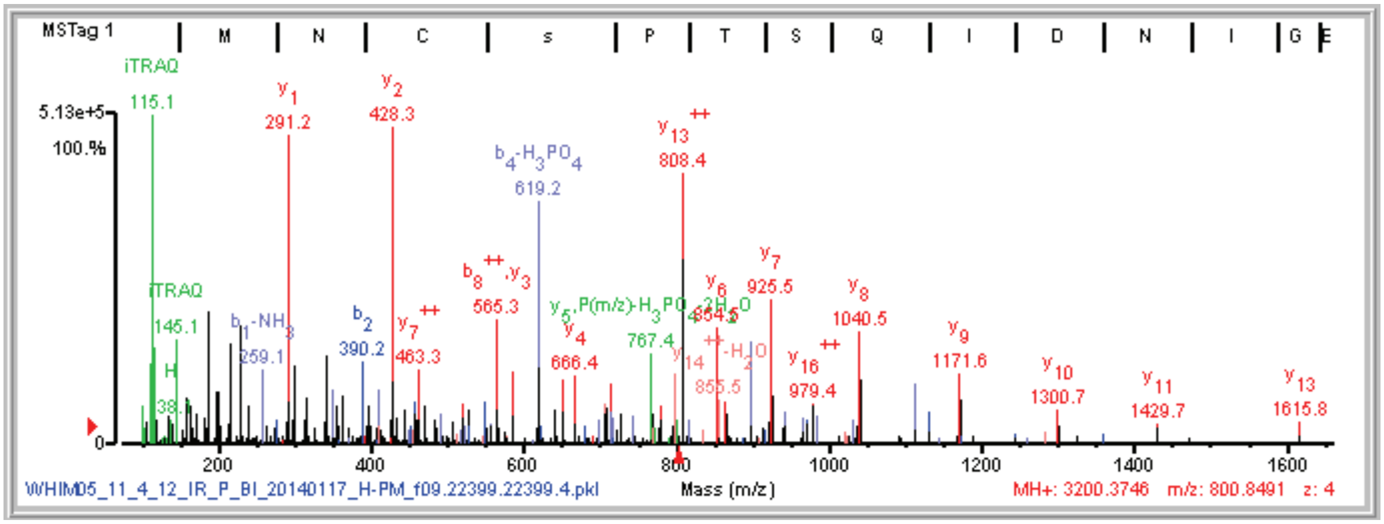

AKT3	S34s	TDGsFIGYK
AKT3	T445t	YFDEEFTAQTItITPPEK
BRAF	S151s	SNPKsPQKPIVR

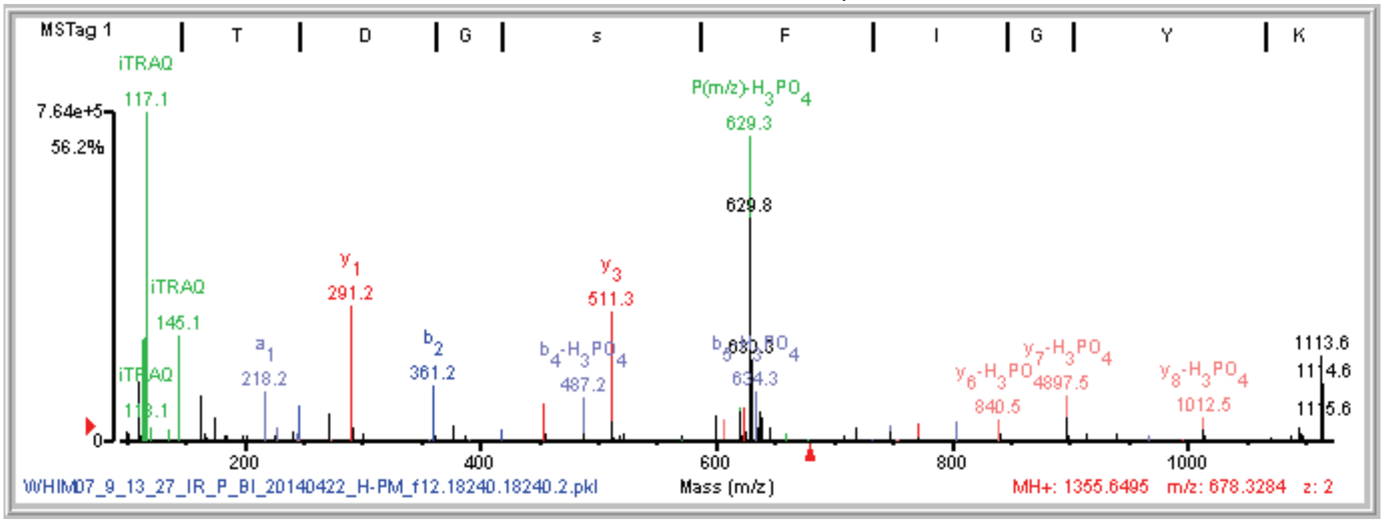

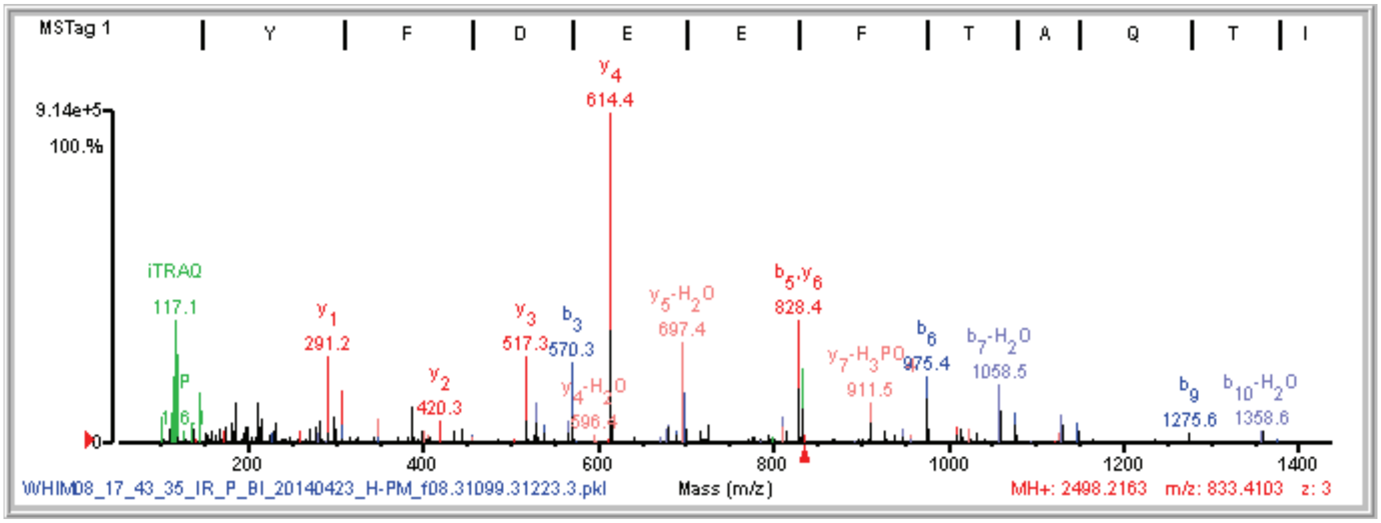

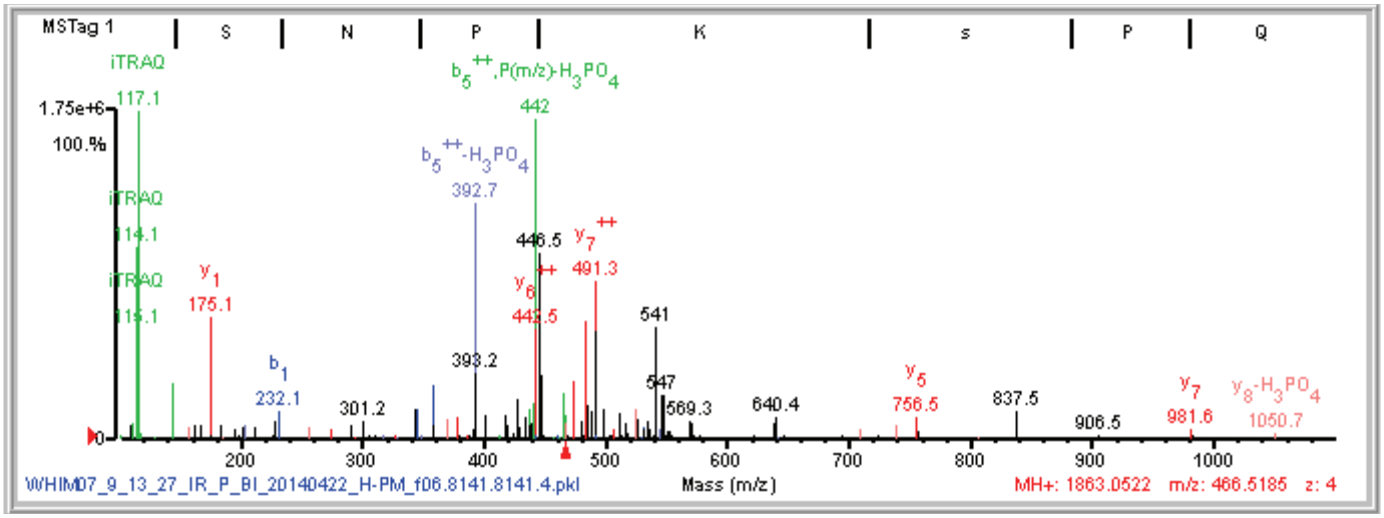

BRAF	S364s	SsSAPNVHINTIEPVNIDDLIR
BRAF	S419s	ALQKsPGPQR
BRAF	S446s	RDsSDDWEIPDGQITVGQR

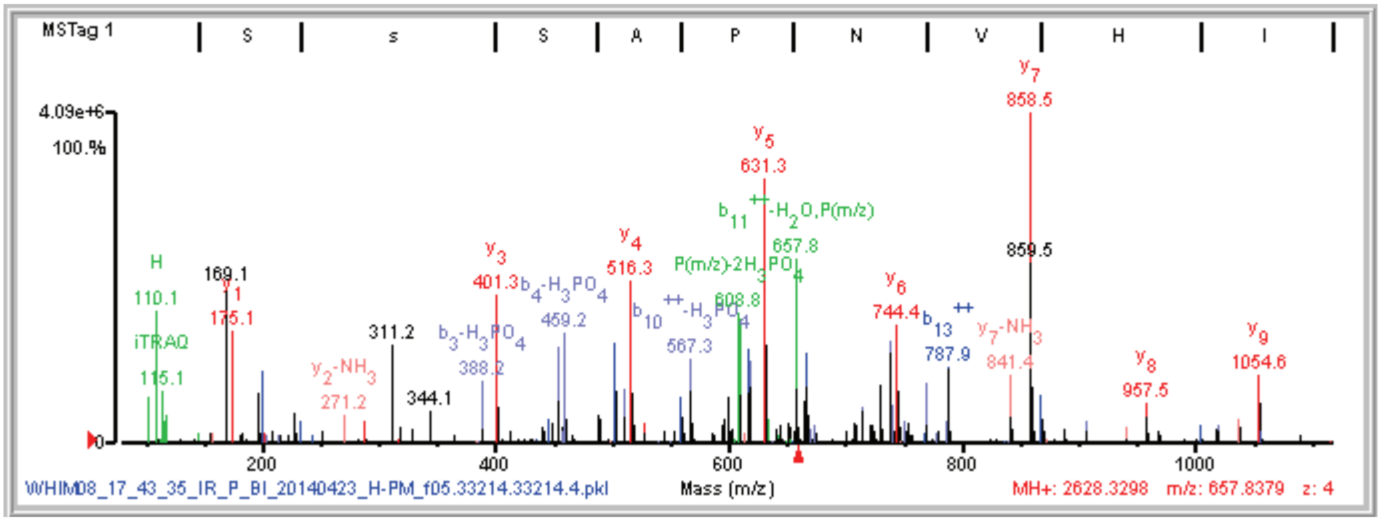

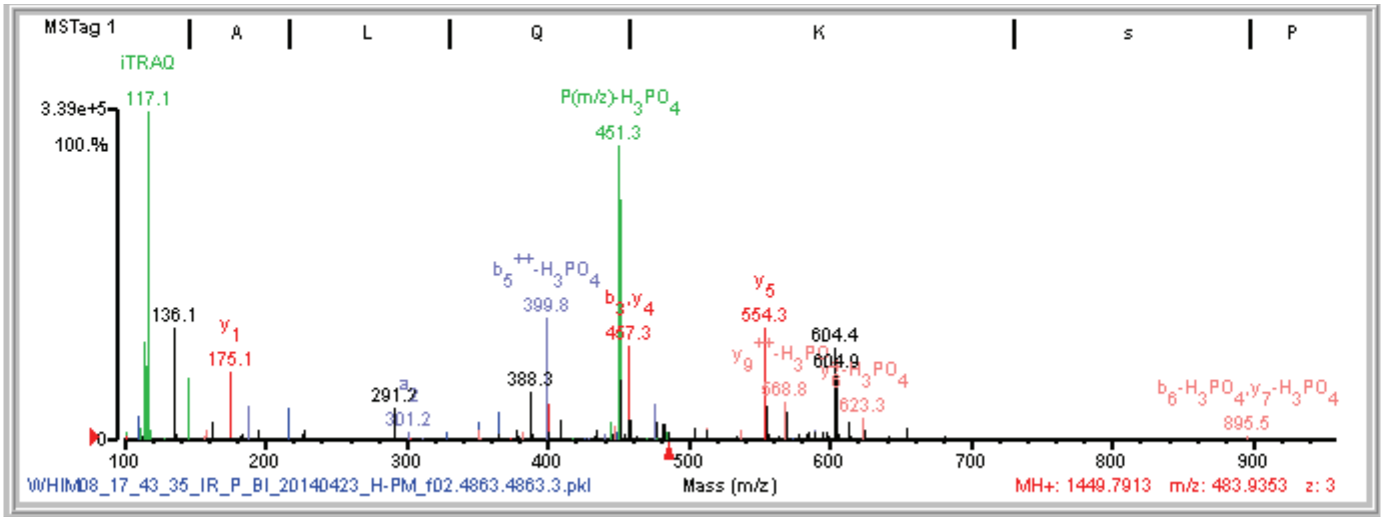

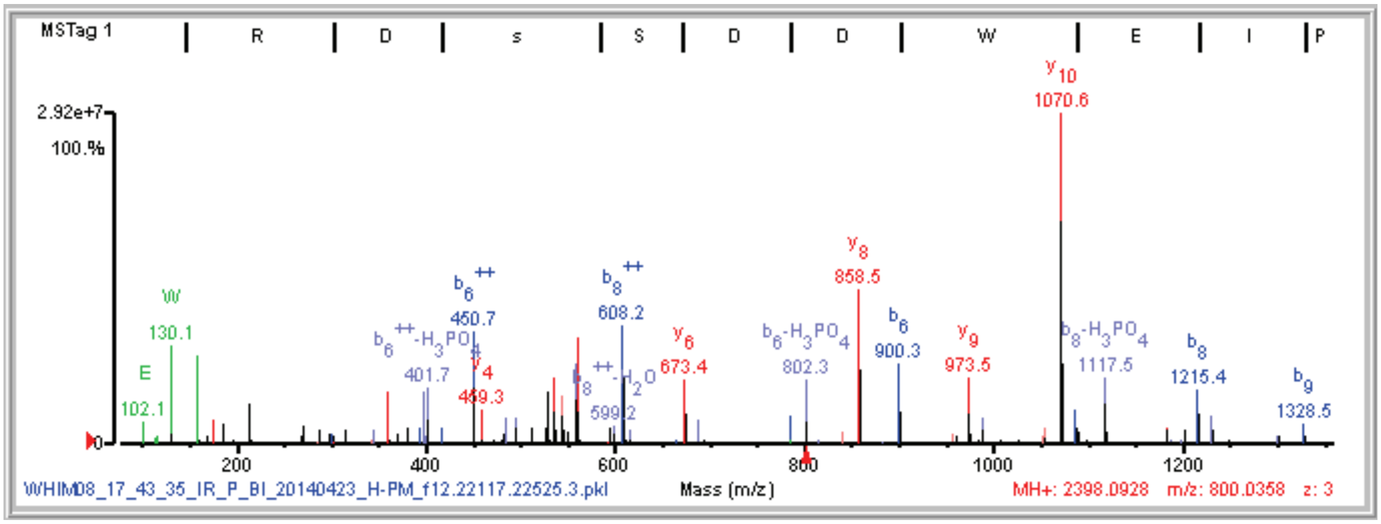

BRAF	S729s	SAsEPSLNR
BRAF	T313t	FFEHHPIPQEEASLAETALtSGSSPSAPASDSIGPQILTSPSPSK
BRAF	T401t	GDGGSTTGLSAtPPASLPGSLTNVK

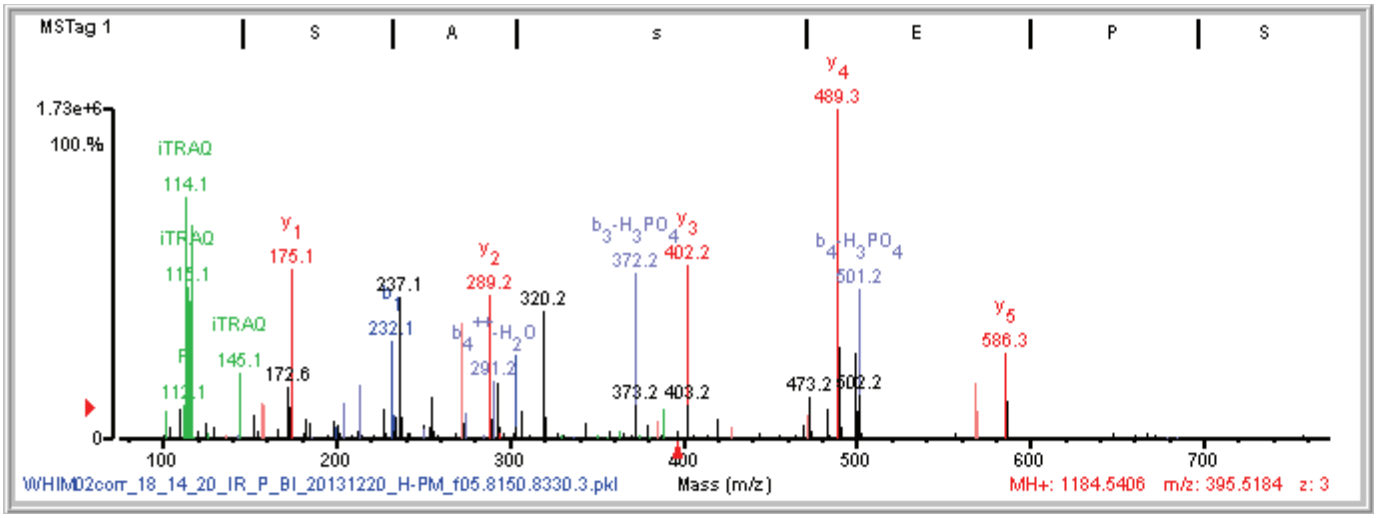

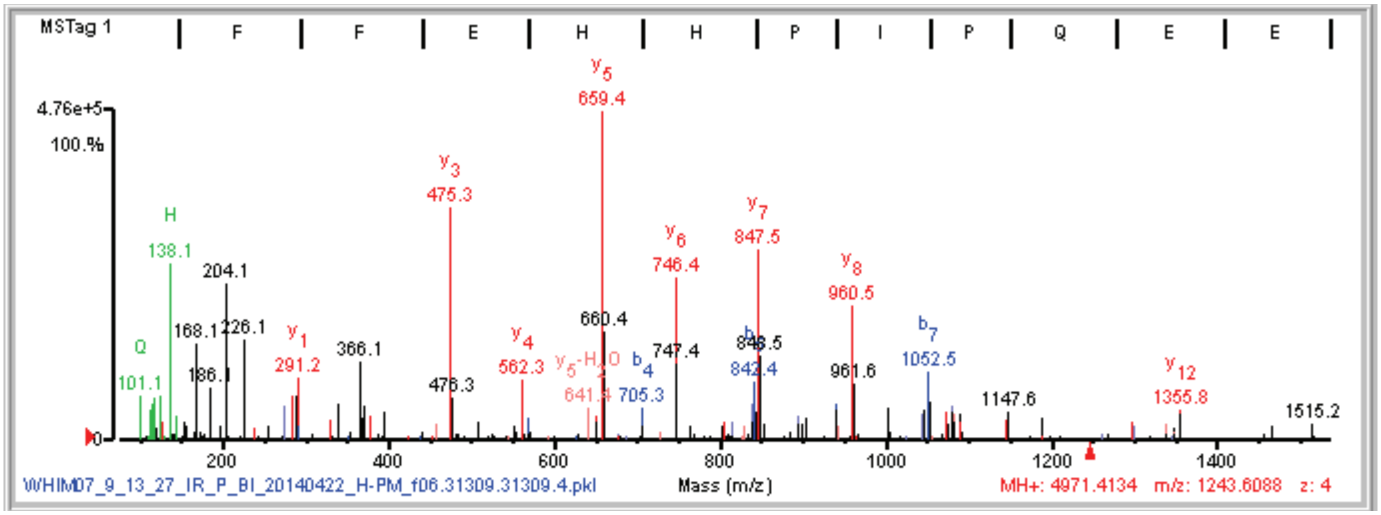

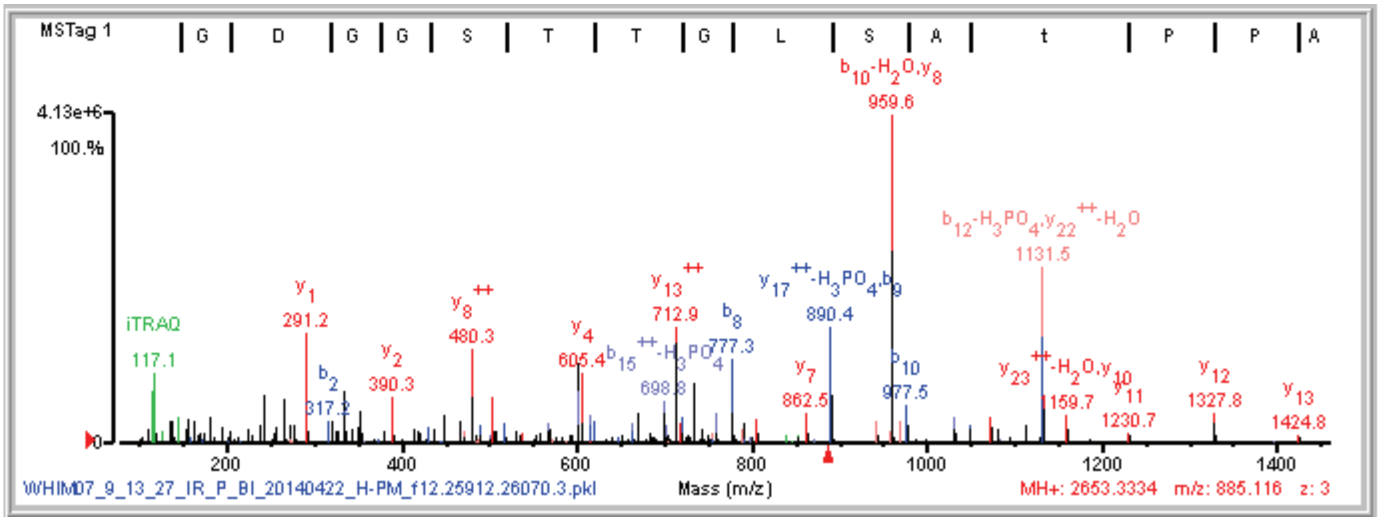

CTNNB1	S29s	AAVSHWQQQsYLDSGIHSGATTTAPSLSGK
ERBB2	S1151s	YSEDPTVPLPSETDGYVAPLTCSPQPEYVNQPDVRPQPPsPR
ERBB2	S728s	VLGsGAFGTVYK

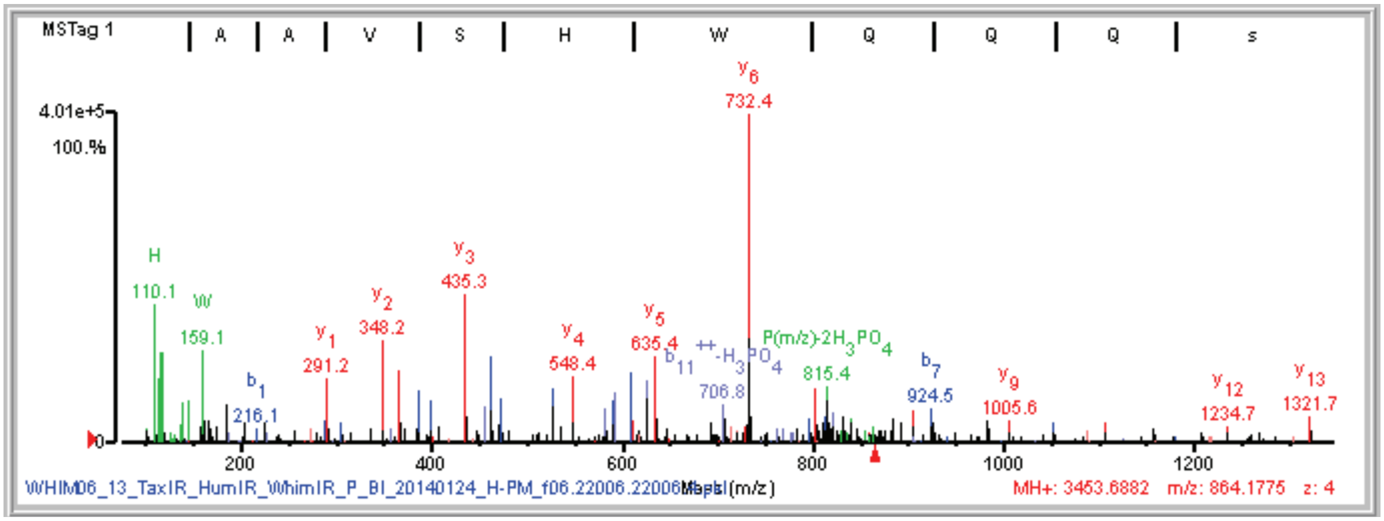

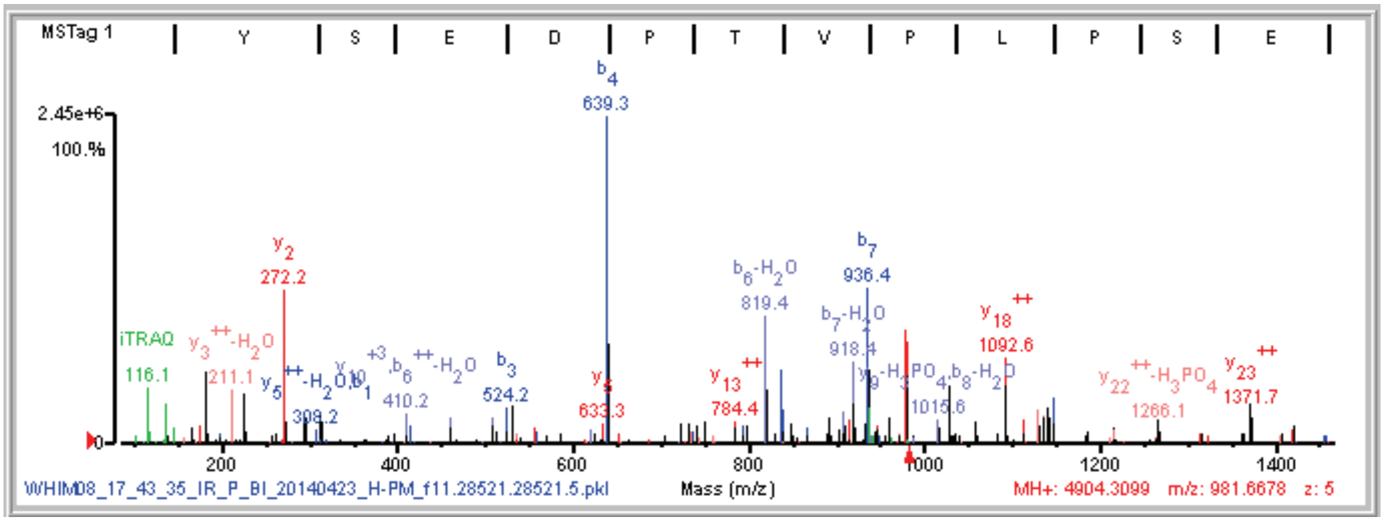

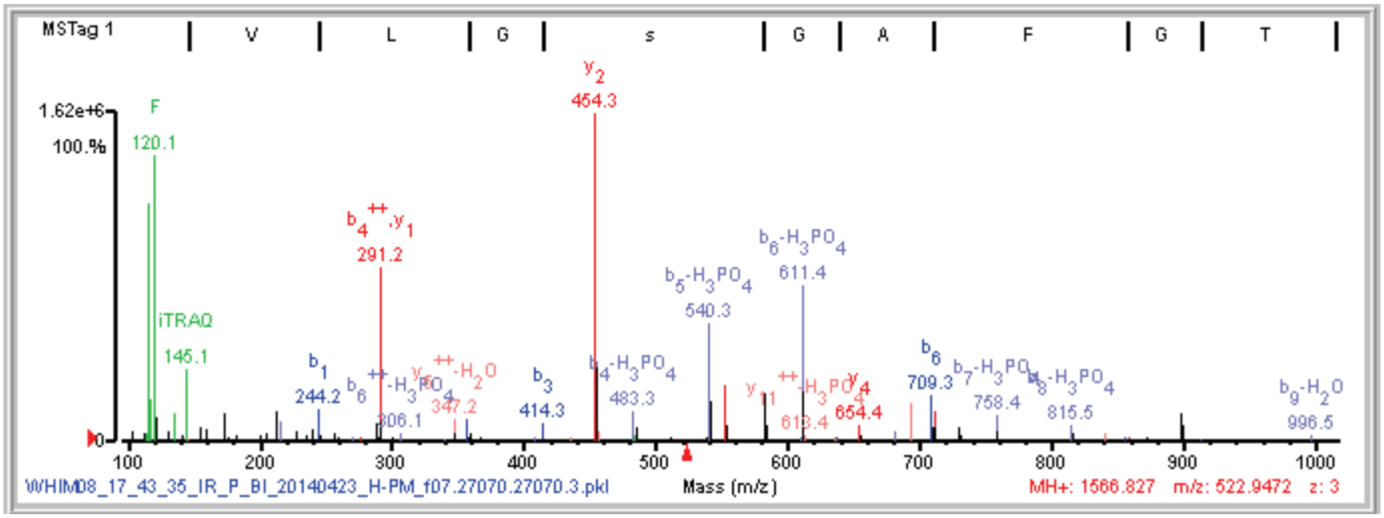

ERBB2	S998s	FVVIQNEDLGPAsPLDSTFYR
ERBB2	T1240t	GtPTAENPEYLGLDVPV
ERBB2	T701t	LLQETELVEPLtPSGAMPNQAQMR

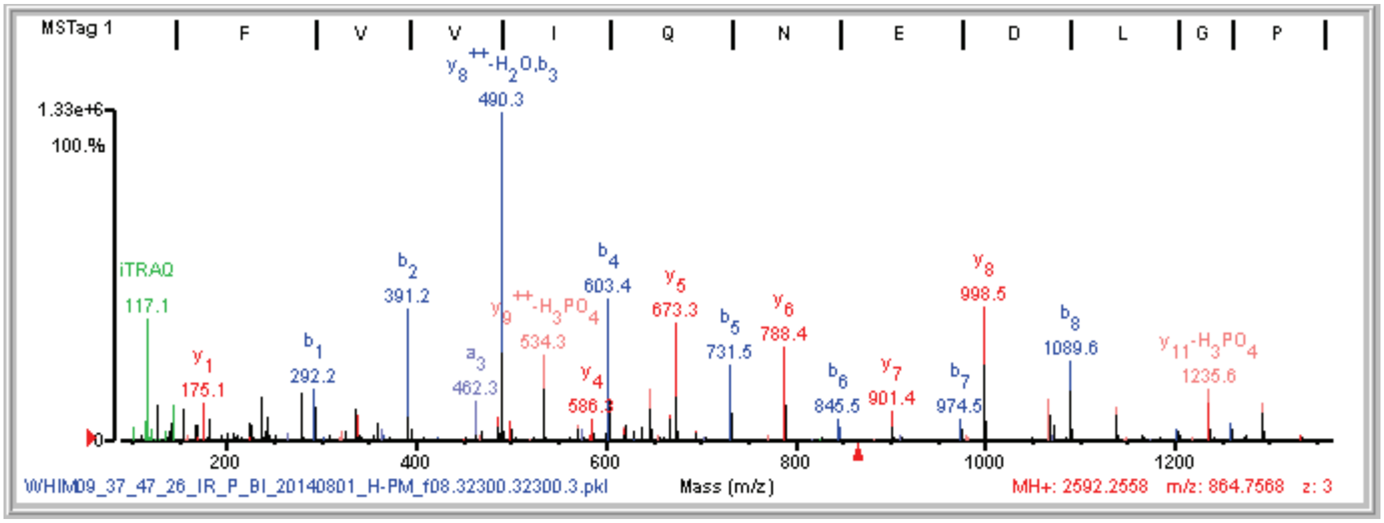

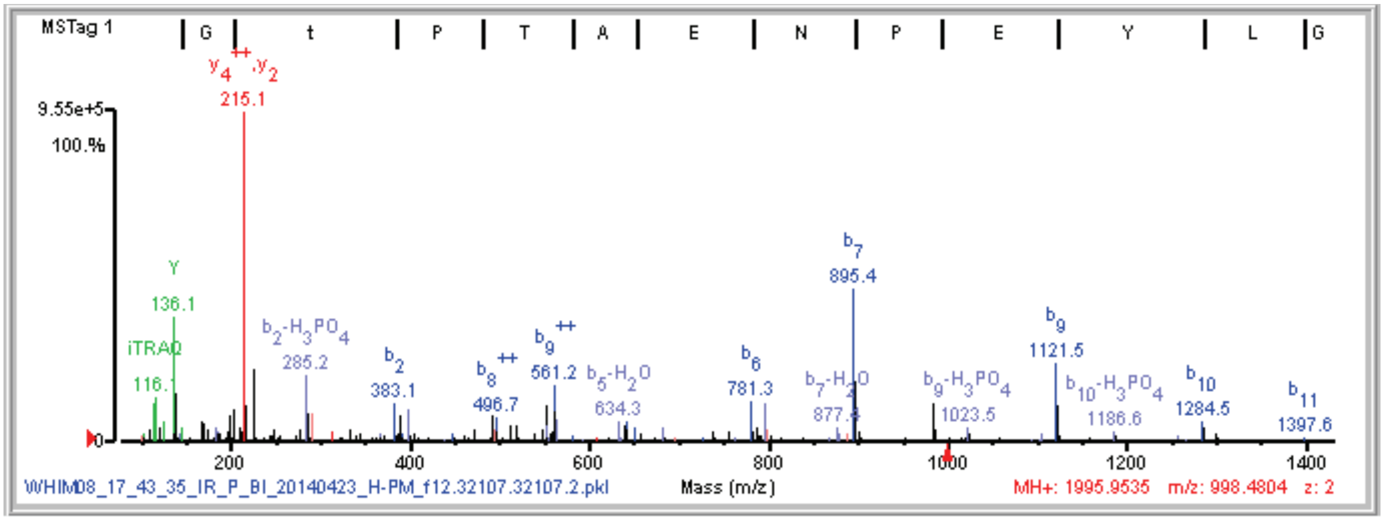

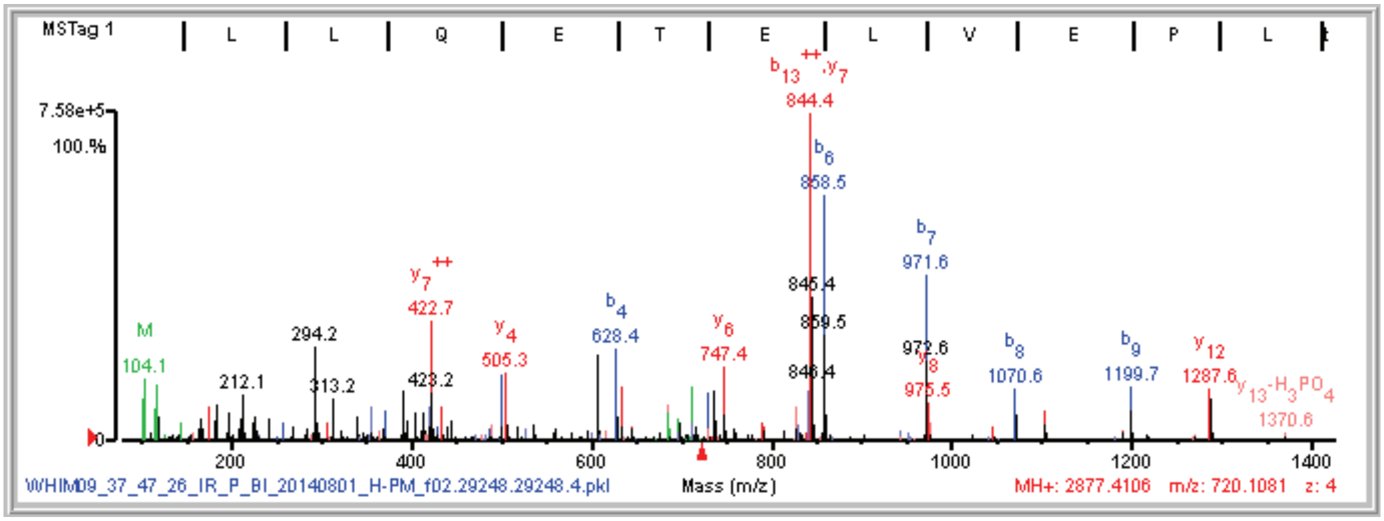

ERBB2	Y1248y	GTPTAENPEyLGLDVPV
FGFR4	S573s	RPPGPDLsPDGPR
HSP90AB1	S226s	EIsDDEAEEEKGEK

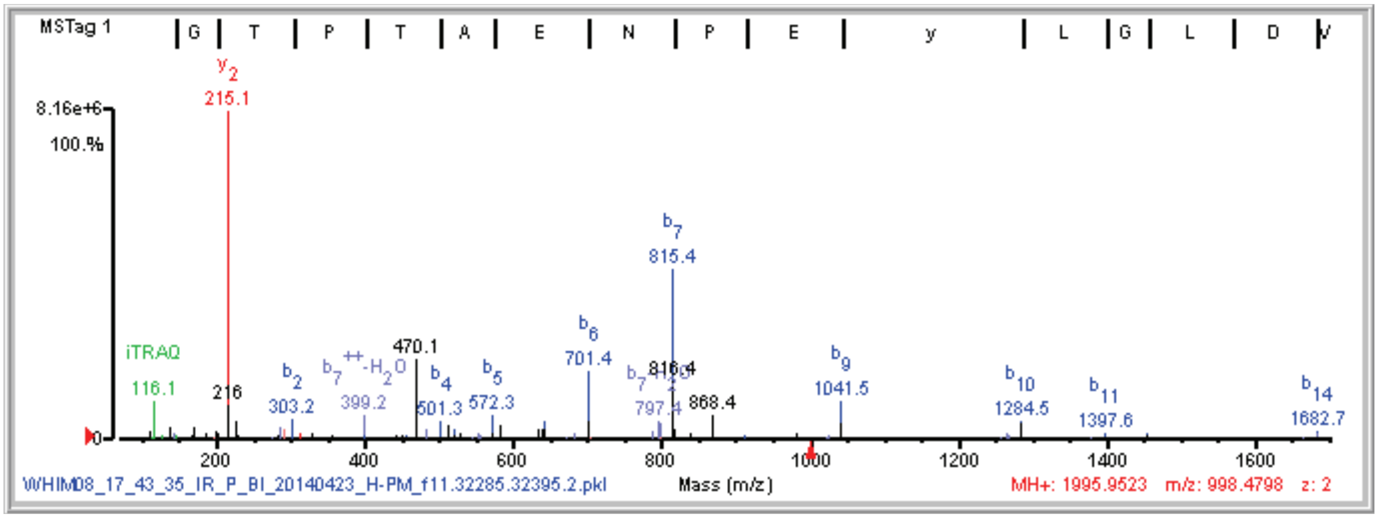

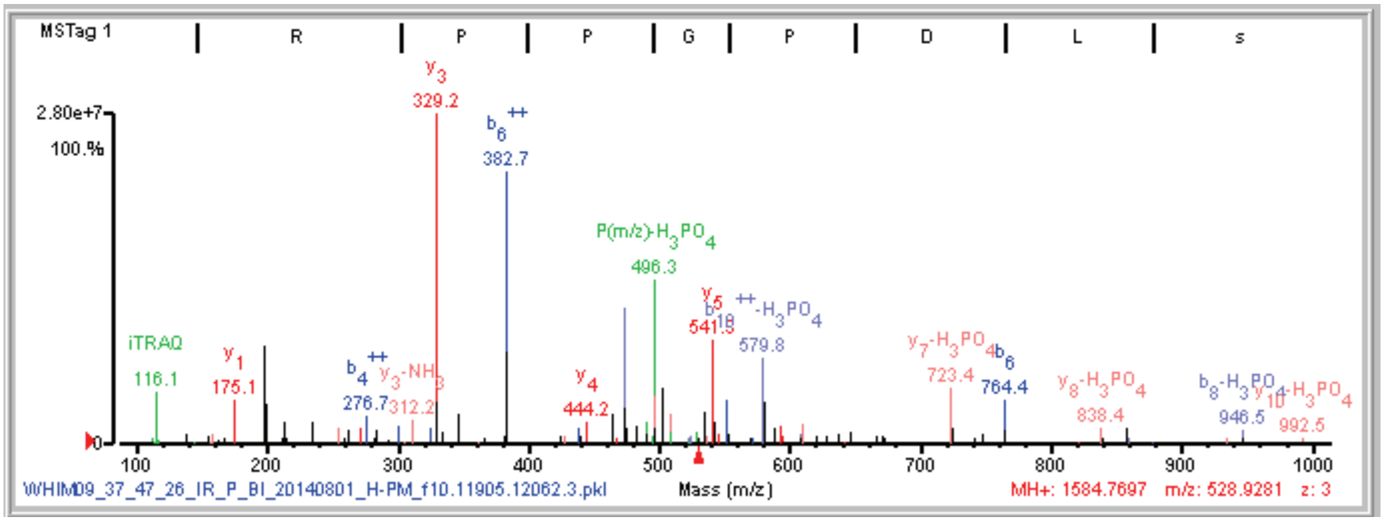

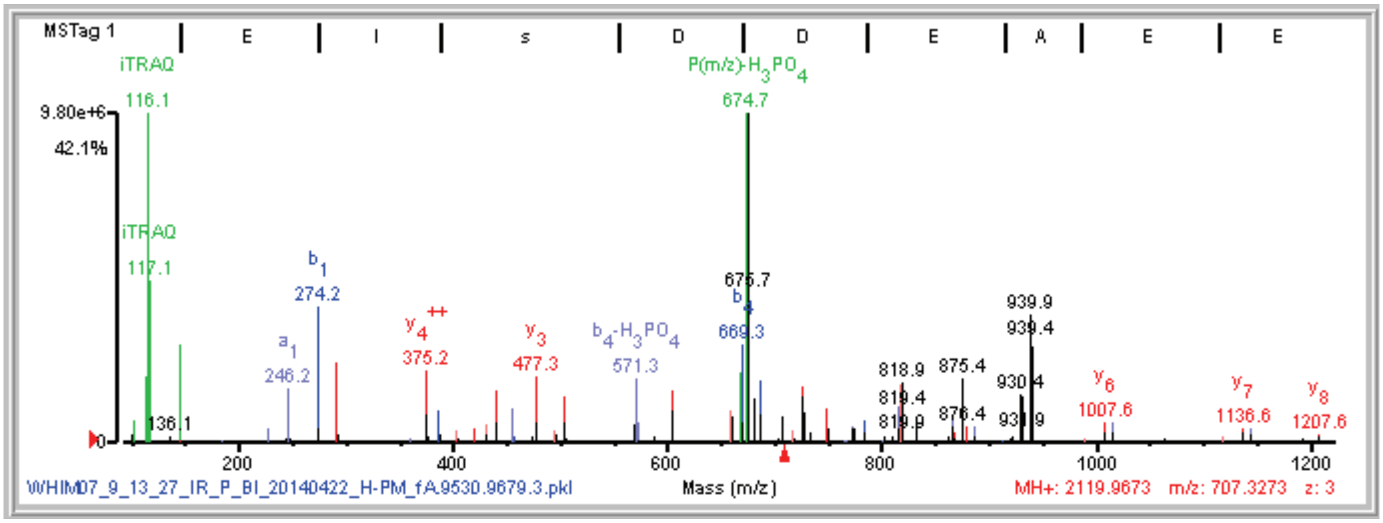

HSP90AB1	S307s	sLTNDWEDHLAVK
HSP90AB1	Y56y	IRyESLTDPSK
HSP90B1	T786t	VEEEPEEEPEETAEDTTEDTEQDEDEEMDVGtDEEEETAK

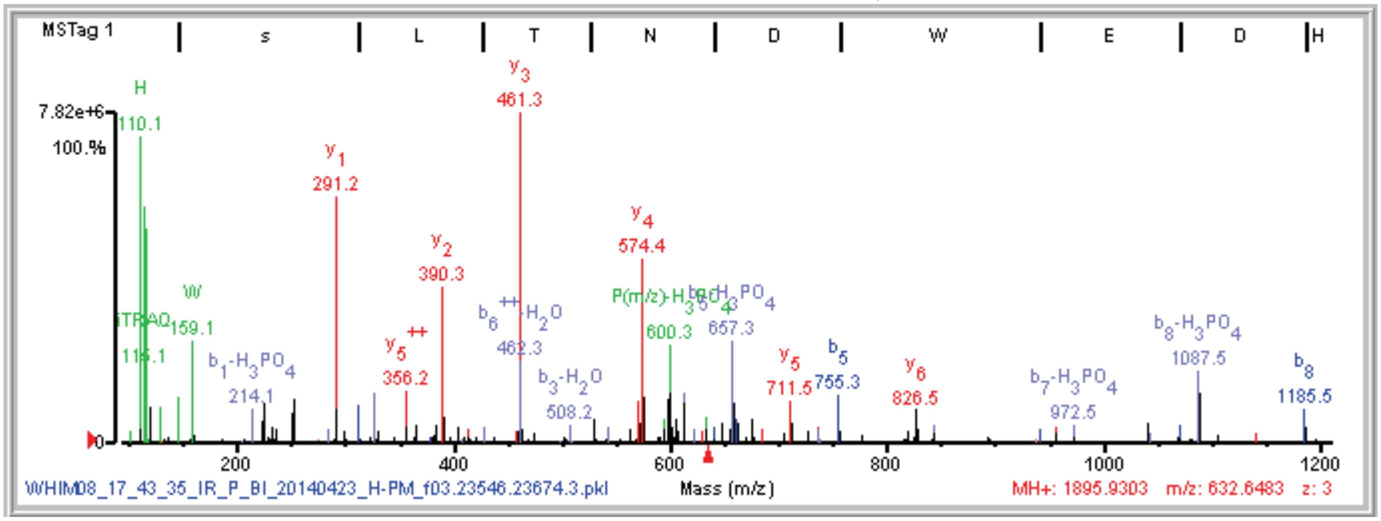

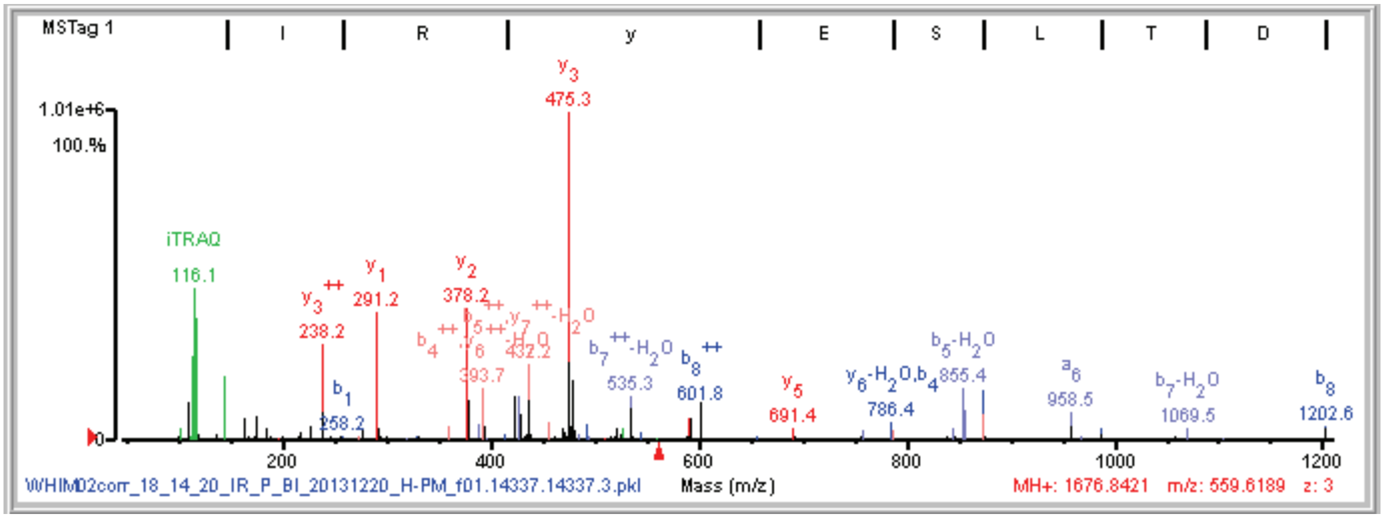

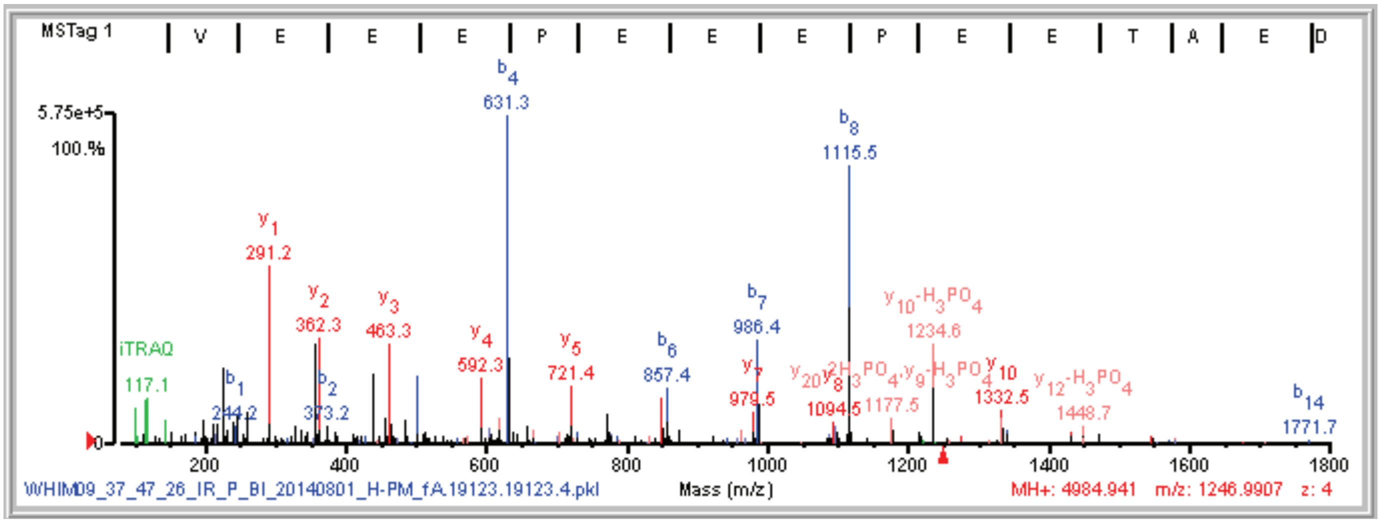

MTOR	S2478s	TGTTVPEsIHSFIGDGLVKPEALNK
MTOR	S2481s	TGTTVPESIHsFIGDGLVKPEALNK

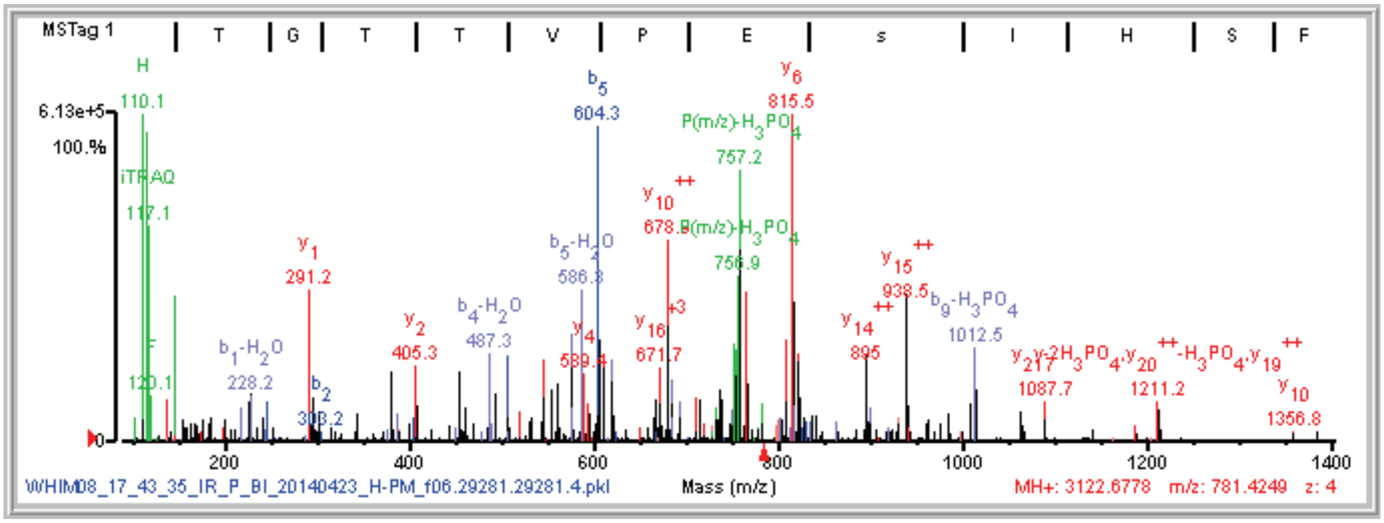

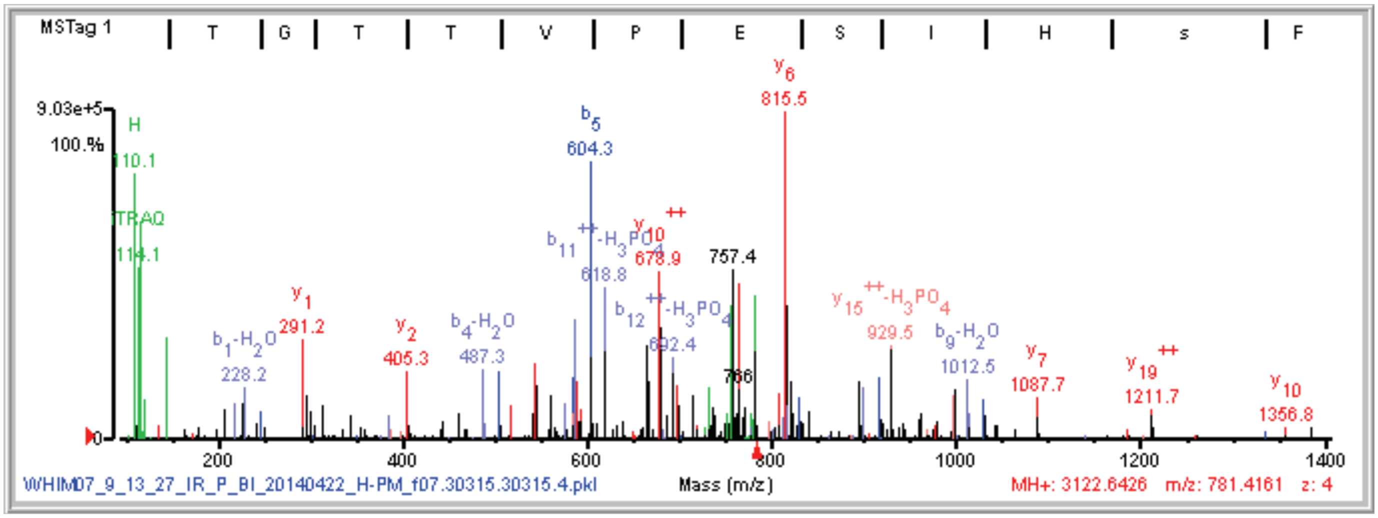

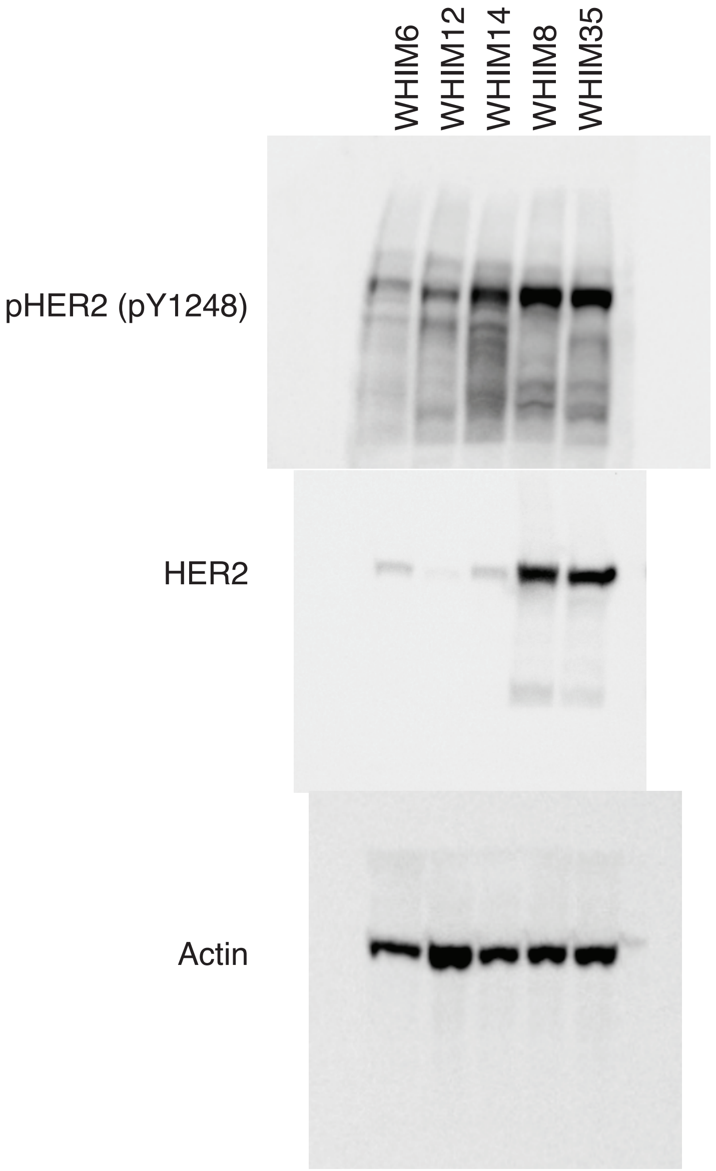

Supplementary Figure 14. Western blots of actin, HER2 protein and HER2 p.Y1248 expression levels in 5 WHIM models (WHIM6, WHIM8, WHIM12, WHIM14, and WHIM35).

a

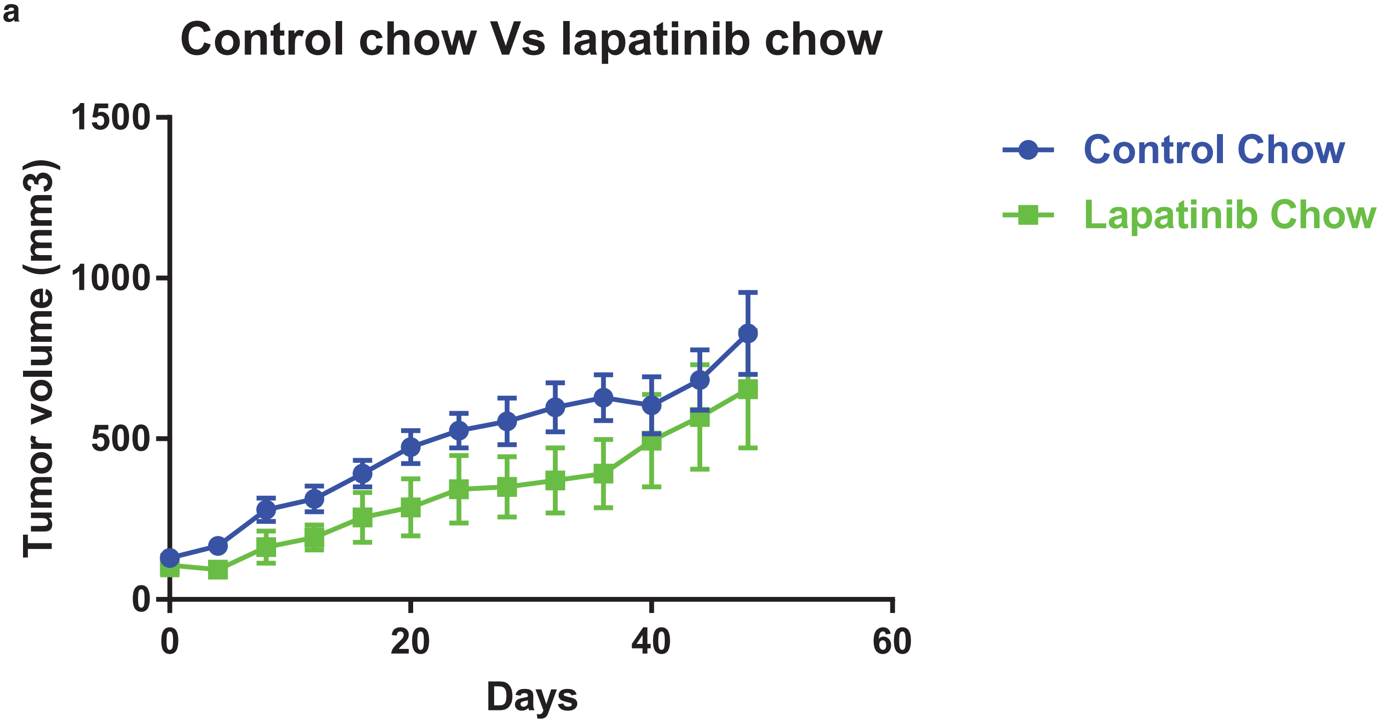

b

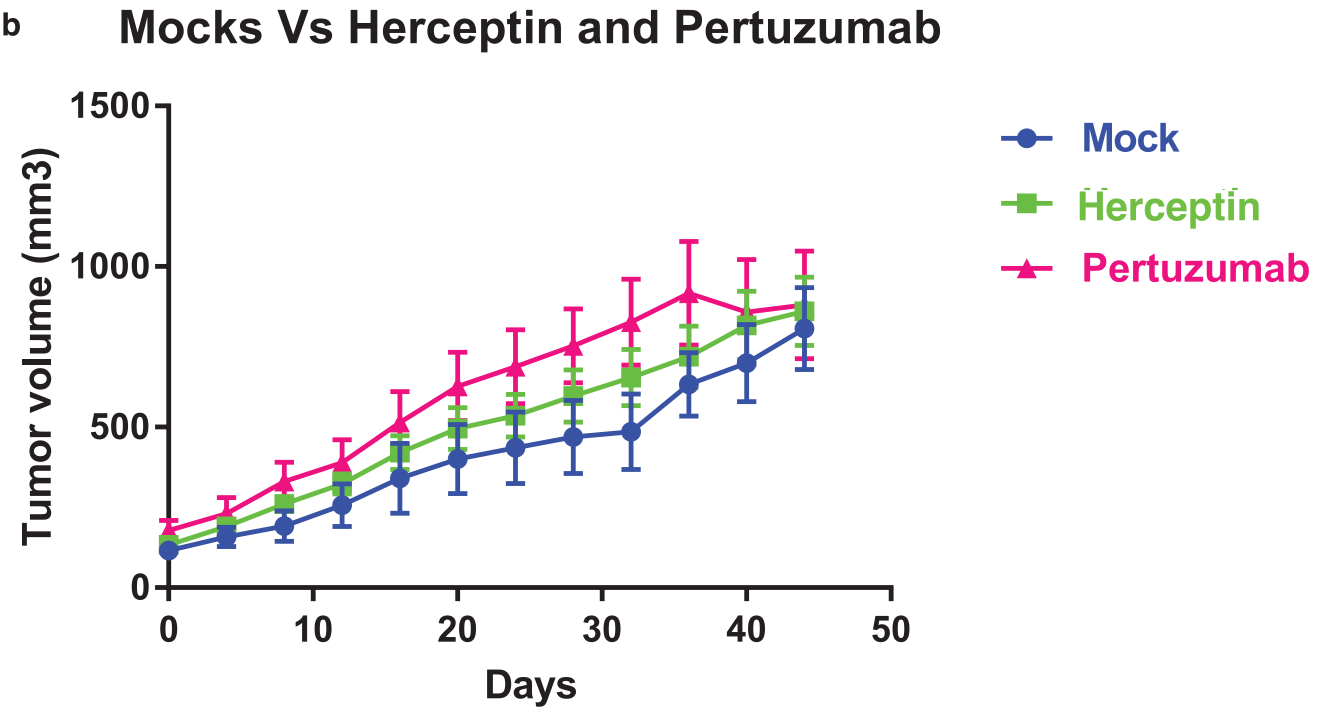

Supplementary Figure 15. Targeted HER2-inhibition treatments in WHIM14. (a) In vivo treatment responses to a lower, clinically achievable dose of lapatinib (30mg/kg). (b) In vivo treatment responses to trastuzumab and pertuzumab. Values were represented by tumor volume [mm3] every 4 day following treatment.
